# An Evolution-Based Approach to *De Novo* Protein Design and Case Study on *Mycobacterium tuberculosis*

**DOI:** 10.1371/journal.pcbi.1003298

**Published:** 2013-10-24

**Authors:** Pralay Mitra, David Shultis, Jeffrey R. Brender, Jeff Czajka, David Marsh, Felicia Gray, Tomasz Cierpicki, Yang Zhang

**Affiliations:** 1 Department of Computational Medicine and Bioinformatics, University of Michigan, Ann Arbor, Michigan, United States of America; 2 Department of Pathology, University of Michigan, Ann Arbor, Michigan, United States of America; 3 Department of Biological Chemistry, University of Michigan, Ann Arbor, Michigan, United States of America; Harvard University, United States of America

## Abstract

Computational protein design is a reverse procedure of protein folding and structure prediction, where constructing structures from evolutionarily related proteins has been demonstrated to be the most reliable method for protein 3-dimensional structure prediction. Following this spirit, we developed a novel method to design new protein sequences based on evolutionarily related protein families. For a given target structure, a set of proteins having similar fold are identified from the PDB library by structural alignments. A structural profile is then constructed from the protein templates and used to guide the conformational search of amino acid sequence space, where physicochemical packing is accommodated by single-sequence based solvation, torsion angle, and secondary structure predictions. The method was tested on a computational folding experiment based on a large set of 87 protein structures covering different fold classes, which showed that the evolution-based design significantly enhances the foldability and biological functionality of the designed sequences compared to the traditional physics-based force field methods. Without using homologous proteins, the designed sequences can be folded with an average root-mean-square-deviation of 2.1 Å to the target. As a case study, the method is extended to redesign all 243 structurally resolved proteins in the pathogenic bacteria *Mycobacterium tuberculosis*, which is the second leading cause of death from infectious disease. On a smaller scale, five sequences were randomly selected from the design pool and subjected to experimental validation. The results showed that all the designed proteins are soluble with distinct secondary structure and three have well ordered tertiary structure, as demonstrated by circular dichroism and NMR spectroscopy. Together, these results demonstrate a new avenue in computational protein design that uses knowledge of evolutionary conservation from protein structural families to engineer new protein molecules of improved fold stability and biological functionality.

## Author Summary

The goal of computational protein design is to create new protein sequences of desirable structure and biological function. Most protein design methods are developed to search for sequences with the lowest free-energy based on physics-based force fields following Anfinsen's thermodynamic hypothesis. A major obstacle of such approaches is the inaccuracy of the force-field design, which cannot accurately describe atomic interactions or correctly recognize protein folds. We propose a novel method which uses evolutionary information, in the form of sequence profiles from structure families, to guide the sequence design. Since sequence profiles are generally more accurate than physics-based potentials in protein fold recognition, a unique advantage lies on that it targets the design procedure to a family of protein sequence profiles to enhance the robustness of designed sequences. The method was tested on 87 proteins and the designed sequences can be folded by I-TASSER to models with an average RMSD 2.1 Å. As a case study of large-scale application, the method is extended to redesign all structurally resolved proteins in the human pathogenic bacteria, *Mycobacterium tuberculosis*. Five sequences varying in fold and sizes were characterized by circular dichroism and NMR spectroscopy experiments and three were shown to have ordered tertiary structure.

## Introduction

Computational protein design aims to identify new amino acid sequences that have desirable 3-dimensional (3D) structure and biological function. This can be considered as a reversed procedure of protein folding and protein structure prediction, in that protein folding and protein structure prediction aim to deduce the 3D structure from given amino acid sequences. In protein 3D structure prediction, it has been well-established [1]–[2] that the most reliable and accurate models are those constructed by homology modeling which copies and refines structural frameworks from evolutionarily related proteins for the template-based modeling targets. The sequence profiles, which scale the evolutionary conservation/mutation characteristics of protein families in a form of a *L*×20 matrix [3], play a central role in improving the alignment accuracy of structural template identifications [4]–[5]. On the other hand, *ab initio* folding approaches, which try to fold proteins using physics-based force fields, work well only occasionally for small proteins (<100 residues) with low resolution (RMSD>3–5 Å) [6]–[8]. The difficulty of the physics-based *ab initio* approaches was generally considered to be due to the inaccuracy of force field design and the limits of the conformational search [9]–[10]. Recently, a super-long time (>100 µs) molecular dynamics simulation by Raval et al [11] demonstrated that the conformational search is a factor of less impact to the failure to protein folding and structure refinement compared to the force field accuracy. Zhang et al [12] and Mirjalili et al [13] further showed that the spatial restraints from structural templates can help improve the energy funnel of the physics-based force field and guide the molecular dynamics simulation for structure refinements. However, these refinements are limited to fine-tuning the local structure details and are far from topology-level improvements.

Somewhat in paradox, the physics-based force field, which has been exploited in most of current approaches [14]–[21], seems to work well on protein designs. A number of newly designed proteins with improved structural stability and/or biological functionality have been reported [14], [22]–[25]. One of the reasons for the success is probably due to the iterative searching simulations, which reinforce the match of the designed sequence with the target structure that can result in a simplified energy landscape of the design sequence. As a result, the folding accuracy of structural models on the designed sequences can be significantly increased compared to that encountered in structure prediction of natural proteins [18], [26]. To further improve the biological specificity of designed sequences, Floudas and co-workers recently introduced constraints from sequence homology search, including charge, amino acid content and residue frequency, to guide the physics-based sequence designs [25], [27]–[29]. Nevertheless, many of the physics-based designs are structurally and thermodynamically less well-defined than natural proteins [14], [30]. Similar to the protein folding problem, one major difficulty stems from the inaccuracy of the force field to balance the subtle atomic interactions and to distinguish the unique structures from alternatives, especially for the medium-to-large size proteins. In addition, the exponential increase in sequence phase space with protein size *L* (∼20*^L^*) is prohibitive for direct design enumerations.

To address these issues, we propose an evolution-based protein design method, whereby sequence space search is constrained by the sequence and structural profiles collected from protein analog families, with local side-chain packing accommodated by neural-network based solvation and secondary structure predictions. The principle of the approach follows the critical lessons that we learnt from threading-based protein structure prediction methods, i.e. to use the reliable “finger print” of nature in the form of structural profile information to guide the simulation to the fold of the target scaffold structure.

To examine the generality of the approach, we compared a combined evolutionary and physics based method (EBM) against a stand-alone physics-based design method [19], termed PBM, on a large set of proteins using computational protein structure prediction methods to test the foldability and physicochemical compatibilities of the designed sequences. As a case study of large-scale applications, the EBM method was extended to redesign all proteins from the pathogenic bacteria *Mycobacterium tuberculosis* (MTb), which is the second leading cause of death from infectious diseases [31]. Finally, a handful of designed domains were expressed, purified and biophysically characterized by circular dichroism (CD) and NMR spectroscopy experiments for various folding feature validations.

## Results

**Figure 1:**
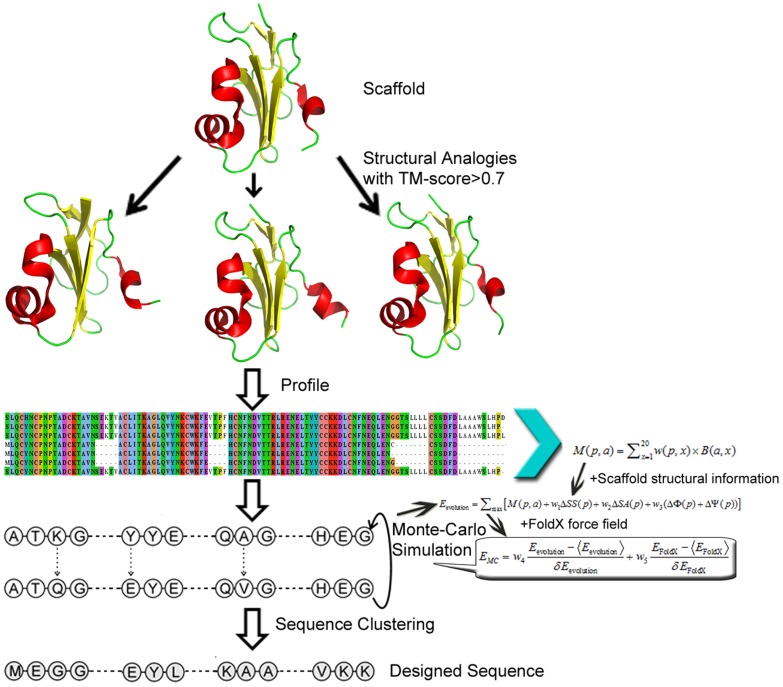
An overview of the evolution-based protein design method (EBM). The procedure consists of profile construction, Monte Carlo search, and design selection.

The outline of our protein design procedure is shown in Figure 1, which consists of structural profile construction and the profile-guided Monte Carlo sequence space searching simulation. The final designed sequence of the lowest free-energy is identified by sequence-based clustering (see Materials and methods). The test set contains 87 proteins randomly collected from the PISCES server [32] with cutoffs of resolution ≤1.6 Å and sequence identity ≤30%. Visual inspection was further performed to retain proteins with globular folds since many proteins of irregular shape are unstable on their own. The length of the proteins varies from 52 to 197 residues. As per SCOP, this set includes 17 alpha proteins (α), 22 beta proteins (β), 14 alpha/beta proteins (α/β), 32 alpha and beta proteins (α+β), and two small proteins with little secondary structure.

### Rational of folding simulations for protein design verifications

To evaluate the likelihood of the designed sequences to fold into stable and desired structures (or the foldability), we exploited the I-TASSER pipeline [33]–[34] to generate structural models for each of the designed sequences and then examine the structural similarity to the target scaffold, where all homologous templates to the scaffold sequence were excluded from the threading structure library. Here, one reason for the choice of the computational folding approach for validation is that experimental validations are generally too expensive for large-scale protein design experiments.

Second, the current *ab initio* folding methods have limited ability to fold protein structures beyond 100 residues. In contrast, the I-TASSER pipeline has a high success rate (∼3/4) to construct correct folds for medium-to-large sized proteins by structurally reassembling the fragments excised from threading template structures without using homologous templates, as demonstrated by the recent community-wide CASP experiments [35]–[38]. In a most recent study of the I-TASSER based design validation [19], it was shown that none of the randomized sequences, even with sequence identity to the target higher than the well-designed sequences and having the same secondary structure propensities as the targets (i.e. obtained by integrating segments cut from other PDB structures that had the same secondary structure), could be folded by I-TASSER to a model below 6 Å to the target structures, with the average RMSD to target being 13.4 Å. For the well-designed sequences with optimized tertiary atomic interactions, however, 77% of cases can be folded by I-TASSER to the models below 2 Å. These data demonstrated that the I-TASSER algorithm is indeed selective to native-like sequences, satisfying the minimum requirement for validating the foldability of protein design by computational structure prediction. The data also confirmed that mere coupling of native sequence identity and secondary-structure propensity does not constitute a native-like foldable sequence.

**Figure 2:**
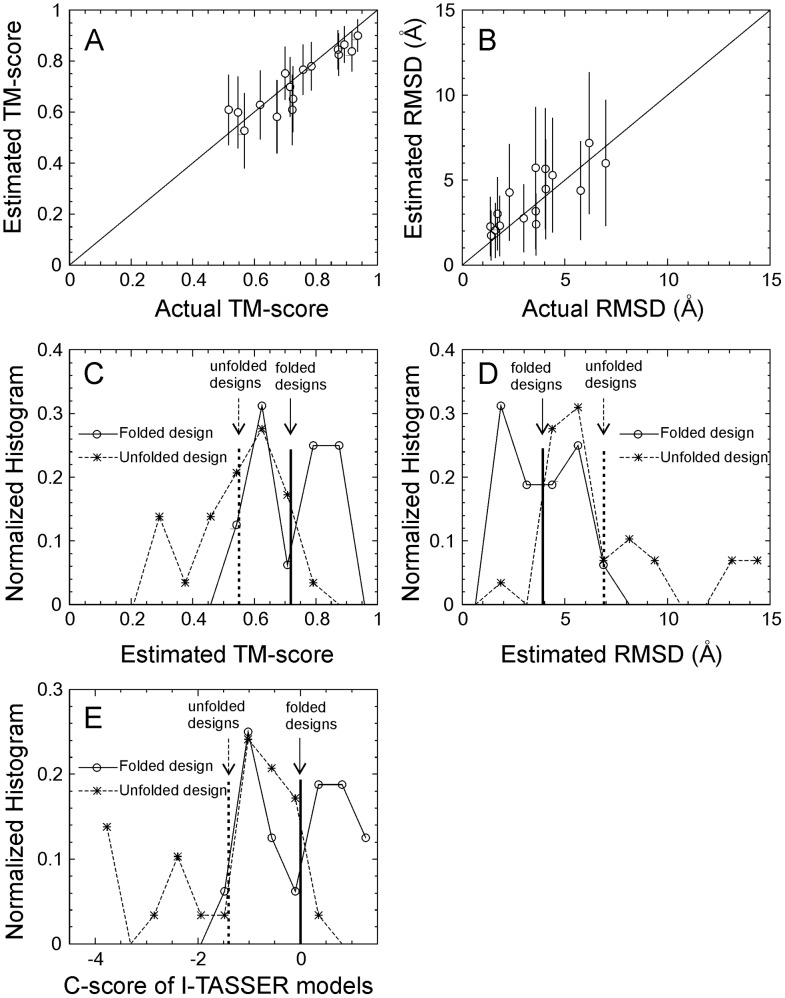
Results of I-TASSER folding on 45 sequences from previous protein design experiments [18], [39]–[51]. (A, B) Estimated TM-score and RMSD of the I-TASSER predicted models versus the actual TM-score and RMSD of the models to the experimental structure for the 16 folded designs. The estimation is calculated based on C-score with an error bar obtained from large-scale benchmark data [52]. (C, D, E) Histogram distributions of estimated TM-score and RMSD, and C-score of the I-TASSER predictions for 16 folded sequences (open circles and solid lines) and 29 unfolded sequences (stars and dashed lines). The vertical lines mark the average values for the folded and unfolded sequences respectively.

For a more realistic test-bed, we collected a set of 45 sequences from previous protein design experiments [18], [39]–[51], which include 16 successful designs with the solved structure deposited in the PDB (folded set) and 29 unsuccessful sequences (unfolded set) defined as “not soluble”, “not folded-CD”, “not folded NMR”, or “natively unfolded”. The unfolded set also includes two of our recent failed designs by EBM on the mouse double minute 2 homolog protein (MDM2) which were experimentally validated as “not folded-CD”, but conceived using a different version of the computational method presented here (Shultis et al, unpublished results). The folded and unfolded sets have a similar average length (98.1 vs. 97.8), with details of the proteins in each set listed in Table S1 of Supplementary Information. Since these proteins have passed various computational feature tests in their designs, these sequences are much closer to real proteins than random sequences. In Figures 2A and 2B, we first ran I-TASSER on the 16 successfully designed proteins and calculated the confidence score (C-score) of each model based on the combination of the threading Z-scores and the convergence of the I-TASSER assembly simulations [52]. For each I-TASSER model, we then estimated TM-scores and RMSD values from the C-score using known correlation equations obtained from large-scale benchmark tests [52]. The data in Figures 2A and 2B show that the estimated TM-score and RMSD of the I-TASSER predictions for the designed proteins are highly correlated with the actual TM-score and RMSD, with a correlation coefficient of 0.91 and 0.80, respectively. The data therefore confirms that the C-score and the estimated TM-score and RMSD values reflect the actual quality of the predicted models, with the actual TM-score and RMSD mostly within the error bars of the estimated values.

In Figure 2E, we applied I-TASSER to both sets of folded and non-folded proteins, where all homologous templates with a sequence identity >25% to the target or detectable by PSI-BLAST were excluded. From Figure 2E, it can be seen that there is an obviously higher percentage of high C-score sequences in the folded design set than that in the non-folded set. The average C-scores are −0.003 and −1.4 for the folded and non-folded sequences respectively (see the vertical lines marked in the Figure). In Figures 2C and 2D, we present the histogram distribution of the estimated TM-score and RMSD calculated on the C-score values for the two sets of sequences. Again, there is a large gap between folded and non-folded sequences, where the average TM-scores (RMSDs) are 0.718 (3.9 Å) and 0.551 (6.8 Å), respectively. In particular, there are much more proteins in the high-quality modeling regions, e.g. with TM-score>0.8 or RMSD<2.5 Å, for the folded sequences than for the non-folded sequences. This data again shows that there is a greater probability of the I-TASSER simulations generating high confidence models close to the target structures for successfully designed sequences than for unsuccessfully designed sequences.

### I-TASSER based 3D structure prediction of designed sequences

In Table 1 (second and third columns), we present a summary of the I-TASSER structural models for the sequences created by both PBM and EBM in comparison with the target structure, where all homologous templates detectable by PSI-BLAST search were excluded from the I-TASSER template library. The average RMSD and TM-score between the I-TASSER models and the scaffold structures are 4.14 Å and 0.74, respectively, for the PBM sequences, while the RMSD and TM-score for the EBM designed sequences are 2.12 Å and 0.87, respectively, which demonstrated an improved foldability by EBM. Here, the average TM-scores are higher than the estimated TM-scores obtained for the sequences taken from the previous design experiments. The major reason is due to the different template filters used in the I-TASSER modeling, since an additional stringent sequence identity cutoff (>25%) was used in the last section. We have confirmed that similar high TM-score values can be obtained when omitting the second homology filter in sequence identity cutoff during the template search. Moreover, as shown in Figure 2A, the estimated TM-score is on average slightly lower than the actual TM-score.

**Table 1:** Evaluation of designed sequences.

Methods	TM-score^a^	RMSD^b^	Normalized relative error (NRE)				Sequence Identity	
			SS^c^	Φ	Ψ	SA^d^	All	Core
PBM^e^	0.74	4.14 Å	2.40	0.43	1.01	0.41	21%	35%
EvBM^f^	0.82	2.82 Å	0.48	0.26	0.30	0.04	27%	35%
EBM^g^	0.87	2.12 Å	0.33	0.14	0.22	0.02	28%	41%

Data is averaged over 87 test proteins. The details on each protein can be found at Table S2, S3, S4.

a TM-score between the first I-TASSER model and the target scaffold.

b RMSD between the first I-TASSER model and the target scaffold.

c SS: Secondary structure.

d SA: Solvent accessibility.

e PBM: Physics-based method using FoldX.

f EvBM: Evolution-based method using only evolutionary terms in Eq. (1).

g EBM: Evolutionary based method using both evolutionary and physics-based terms in Eq. (3).

A detailed analysis on the EBM designed sequences indicates that 80% of the predicted structures have an RMSD of <2.0 Å to the target scaffold, and 42.5% are highly accurate with a RMSD<1.0 Å. For the PBM category, only 54% of the predicted structures have an RMSD<2.0 Å, and 31% have an RMSD<1.0 Å. Having in mind that both the structural profiles and the FoldX potential [53], which were used to design the EBM sequences, are independent from the I-TASSER folding force field, such a high structural similarity between the I-TASSER models and the target structures indicates that the design algorithm should have captured the features essential to the global fold of the target scaffolds.

In Tables S2 and S4, we list the detailed results of the I-TASSER models for each of the testing protein targets by EBM and PBM, respectively. Compared to the EBM designs, the distribution of TM-scores of the PBM designed sequences is more divergent, i.e. the TM-scores are either very high (>0.85) or very low (<0.35), demonstrating that the purely physics-based design is less reliable than the combined physics and evolutionary based EBM approach in designing protein folds. For all 14 cases where the PBM sequences have a low TM-score (<0.3), the combined physics and evolutionary based EBM method drastically improved the TM-scores to >0.75 except in 2ZXY_A (with TM-score from 0.20 to 0.69). This data highlights the efficiency and robustness of the evolutionary profiles in the design of protein folds, which is consistent with observations from protein structure predictions where profile-based threading approaches have been shown to be much more accurate and reliable than physics-based force fields in recognizing protein folds [1], [8].

In Column 4 and 5 of Table S2, we also show the TM-scores of the I-TASSER models to the structural analogs in the profiles and to the scaffold, respectively. On average, the TM-score of the EBM design to the scaffold is 22.5% higher than the TM-score to the structural analogs in the profile. The higher similarity of the I-TASSER models to the scaffold than the structural analogs is probably due to the fact that the scaffold is normally located at the center of the structural analog family, since it was used as the probe for the profile construction. Thus, the consensus effect from the profiles tends to drive the design simulations toward the center structure rather than individual analogs, although all the analog sequences contribute to the consensus effect.

Noteworthily, the average sequence identities between EBM designs and the target scaffold is 28%, which is higher by seven percentage points than the average sequence identity between the PBM designs and the scaffold (21%). This data may raise a question on whether the improved folding accuracy from I-TASSER is just due to the increase in the sequence identity. In our previous study [19], we have demonstrated that the mere coupling of high sequence similarity and secondary structure from random sequences cannot constitute a reasonable rate of I-TASSER folding. Here, we conduct a similar experiment on this set of 87 proteins which randomly generates artificial sequences for each target but with the identity of the artificial sequences to the target being the same to the designed sequences by EBM and PBM, respectively. When we submit the two sets of artificial sequences to the I-TASSER pipelines, both generate non-foldable models of the similarly high RMSD (∼9.7 Å) to the scaffold, which is 4.6 times higher than that of the EBM design sequences.

We have further examined the artificial sequences with a set of more stringent constraints, i.e. with the conserved residues copied from the designed sequences and with the non-conserved residues randomly generated but having a similar residue type (non-polar, polar-uncharged, and charged) as the target sequence (see below for the definition of conserved residues). The secondary structures of the artificial sequences from PSSpred predictions [54] are confirmed to be similar to the target with a Q3 score >70%, i.e. at least 70% of residues having the same secondary structure type (helix, strand or coil) to the target. By including such constraints, the I-TASSER models of the artificial sequences show much closer similarity to the target, with the average RMSD equal to 5.65±2.1 Å for the sequences with the same sequence identity as the EBM proteins and to 5.72±2.2 Å for the sequences having the same sequence identity as the PBM proteins. Nevertheless, the RMSD values of both sets are still significantly higher than the RMSD values of the designed EBM sequences. Despite the difference in sequence identity, the difference in RMSD of the I-TASSER models between the two sets of artificial sequences is negligible compared to the standard deviation. This data again shows that higher sequence similarity in the absence of protein design does not guarantee the significantly more accurate foldability by prediction simulations, or that the drastic improvement in the folding accuracy of EBM sequences (2.12 Å vs. 4.14 Å) should not be attributed to the increase in sequence identity with the EBM method.

### Secondary structure assignment

To examine the secondary structure (SS) distribution of the designed sequences, we developed a new SS prediction method, PSSpred, which combines 7 neural network predictors trained on different PSI-BLAST profiles [54]. In a large-scale test on 3,128 proteins, PSSpred achieves a Q3 accuracy of 84.5%, which is 3% higher than the widely-used PSIpred program [55]. The fourth column of Table 1 shows the SS assignment results of the designed sequences by PSSpred, in comparison to the DSSP assignment on the target structures [56]. To count for the inherent inaccuracy of PSSpred predictions, we calculate the normalized relative error: *NRE* = (*EDS*−*ETS*)/*ETS*, where EDS is the PSSpred prediction error to DSSP on the designed sequence and ETS is that on the target sequence. The NRE of designed sequence by the EBM is 0.33, which is seven times lower than that by the PBM method (column 4, Table 1).

**Figure 3:**
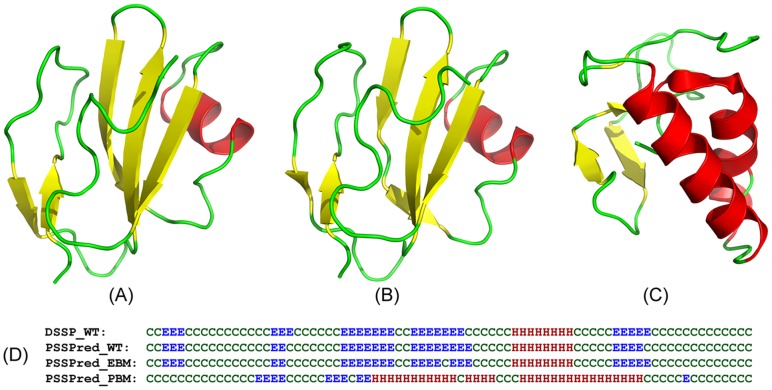
Illustration of protein design on the soluble human CD59. (A) X-ray structure of the target protein. (B) I-TASSER model on the EBM designed sequence. (C) I-TASSER model of the PBM designed sequence. (D) Secondary structure of the target assigned by DSSP, in comparison to that predicted by PSSpred on the target (PSSPred_WT), the EBM (PSSPred_EBM), and the PBM designed sequences (PSSPred_PBM). ‘E’ stands for sheet, ‘H’ for helix and ‘C’ for coil.

In Figure 3, we showed an example of the designs from the soluble human CD59 protein [PDB ID: 2J8B]. The crystal structure of CD59 possesses five beta-strands and one helix packed in a sandwich fold following the DSSP analysis. All the secondary structure elements are present in PSSpred predictions on the target sequence and the EBM based designed sequences (Figure 3D). However, in the sequence designed by the physics-based force field, three strands are completely missed and instead one more long-helix has appeared in the PSSpred prediction on the PBM sequence. Overall, the Q3 accuracy of the PSSpred predictions is 96% for both the EBM sequence and the target sequence, but the Q3 accuracy of the PBM sequence is only 53%, relative to the DSSP assignment of the crystal structure. As a result, I-TASSER folds the EBM sequence to a structural model of RMSD = 1.16 Å to the target (Figure 3B), where the I-TASSER model on the PBM sequence is 9.2 Å away from the target (Figure 3C).

The reduction in the secondary structure error for the EBM method is mainly due to the smoothening effects introduced by the structural profiles and the single-sequence based SS energy terms, which significantly increase the short-range residue cooperation that are usually missed in the physics-based force fields. These effects thus improve the cooperation of secondary structure propensity and the overall foldability of the designed sequences.

### Backbone torsion angle and solvation assignments

To examine the torsion angle and solvation distributions of the residues in the designed sequences, we submitted the designed sequences to sophisticated neural-network predictors [57]–[58]. As shown in Columns 5–7 of Table 1, the normalized relative errors on Φ, Ψ and solvent accessibility (SA) of the EBM designed sequences, relative to the DSSP assignments on the target structures, are 3.1, 4.6 and 20.5 times lower than the corresponding errors for the PBM sequences. Using the same example of the soluble human CD59 in Figure 3, the SA assignments on all the residues are highly consistent with the assignments by DSSP on the target structure, with a Pearson correlation coefficient = 0.74. If we turn off the structural profile restraints, the Pearson correlation coefficient of the designed sequences rapidly reduces to 0.42. Accordingly, for this example the NRE on torsion angle (Φ/Ψ) of the designed sequence increases from 0.04/0.15 to 0.49/0.73. Most of the Φ/Ψ errors are found to occur in the loop regions but many also occur in the regular secondary structural regions, which influences the folding stability of the designed sequences as demonstrated by the high RMSD of the I-TASSER simulations.

### Target sequence recapitulation

In general, a reasonably designed sequence should recapitulate most of the target sequence amino acid identities. However, a high recapitulation rate is not always necessary to guarantee the correct fold and desire function, since a similar fold can be adopted by a variety of protein sequences and families (e.g. the Tim beta/alpha-barrel fold is taken by 33 superfamilies of variant sequences in the SCOP database). In our test set, when using the physics-based FoldX potential, the sequence identity of the designed sequence to native is 21% (35% in the core regions) (Columns 8–9 of Table 1). When the structural profiles are considered, the sequence recapitulation increases only slightly by 6–7%, i.e. 28% in the whole sequence and 41% in the core region. Considering the identity over whole protein sequence, our EBM designs are comparable with Kuhlman et al (27% sequence identity) [59] but lower than Saunders et al (37%) [60]. In the core regions, however, the native repetition for these methods consistently increased to 51% and 57% respectively, which are significantly higher than our designs.

Despite the low sequence recapitulation, the high similarity of the I-TASSER models of the designed sequences to the scaffold structures as reported in Table 1 is striking. To have a better understanding of the implications, we examined the distribution of the recapitulated residues, especially on the evolutionally conserved residues which are often critical to gauge the global fold [61]. For this purpose, we define conserved positions based on the PSI-BLAST sequence family search, i.e. a residue is named as “conserved” if the entropy of the residue (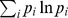) in the multiple sequence alignment of PSI-BLAST search is low (<−0.3). Based on the experimental data in the PDB library, 56% of these conserved residues are located in, or spatially close (<6 Å) to, the functional sites and/or the ligand-binding pockets, which further confirmed the biologically importance of the residues.

We found that 32% of residues in the conserved positions in the EBM sequences are identical to that in the target and 44% of residues in the conserved regions are highly homologous (with a BLOSUM62 mutation score>0.5) to the corresponding residues in the target. In contrast, these percentages are significantly lower (23% and 29%, respectively) for the PBM sequences. These data show that the structural profiles generated in the EBM pipeline help recognize the highly conserved residues in the evolutionary protein families, which are essential for retaining the protein global fold and biological functionalities. As demonstrated in the I-TASSER folding results, these additional conserved residues facilitate the identification of the better quality of structural fragments and frameworks, which are essential to the correct modeling of the global fold of the proteins (Table 1).

### Amino acid composition

**Figure 4:**
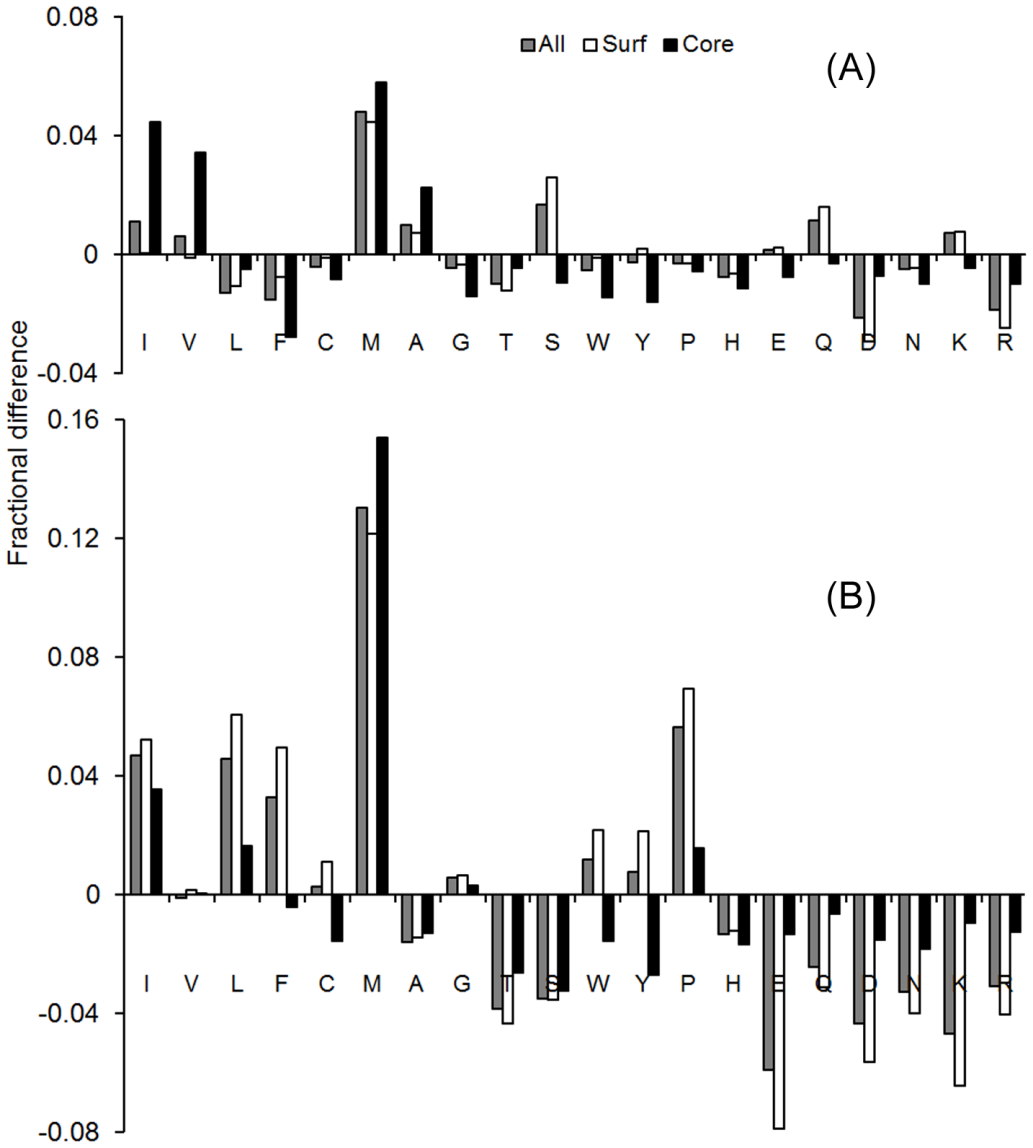
Average fractional difference in amino acid composition between the target and designed sequences. (A) EBM; (B) PBM.

Despite the relatively low sequence identity, we found that the amino acid composition and solvation propensity are similar to the target protein. Figure 4 presents the average difference in the fraction of amino acid composition between the designed and target sequences. Here, a positive value indicates a preference for a particular amino acid in the designed sequences over the target sequence, and vice versa for a negative value. Amino acids are plotted from left to right in order of decreasing hydrophobicity. To have more insight into the distribution of amino acid on the 3D structure, residues are further divided into the core and surface regions based on solvent accessibility, i.e. an amino acid is considered to be at the core region if the relative accessible surface area is <0.16; otherwise it is on the surface.

The EBM derived sequences show a relatively even distribution of amino acids irrespective of their hydropathy scale (Figure 4A). The overall absolute composition difference from the target proteins is 1.1%. In comparison, the composition difference with the PBM sequences is ∼3 times larger with an average deviation 3.4%. An obvious trend in the PBM sequences is the preference of hydrophobic amino acids over hydrophilic, which were also observed in previous physics-based designs [19], [24]; this is mainly due to the biasness of physics-based potential (e.g. FoldX function [53]) towards hydrophobic residues for the stability of protein. In particular, the PBM mode leads to consistent over-enrichment of Methionine and Proline. This feature may disrupt regular secondary structure elements due to the exceptional conformational rigidity of the amino acids [19].

### Number of structures needed for structural profile construction

The structural folds in the PDB library are highly uneven and therefore not all target structures have a sufficient number of analogs. An important issue to the EBM design is to examine how the performance of designed sequences depends on the number of available structural analogs.

**Figure 5:**
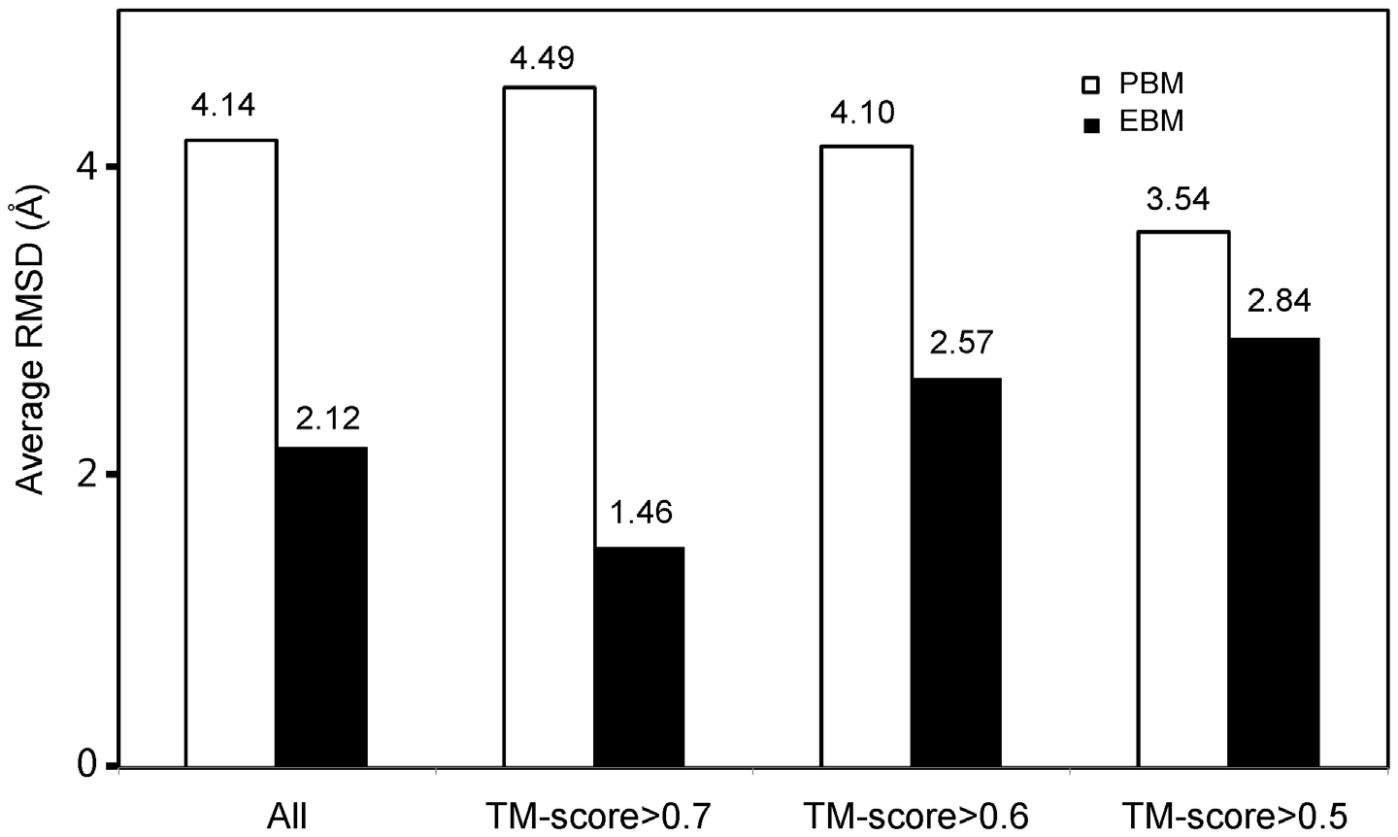
Average RMSD between the scaffold structures and I-TASSER models on the proteins designed by PBM and EBM. The dataset is divided by the TM-score cutoff of the template proteins that were used for constructing the sequence profiles.

In our EBM design pipeline, we set a default cutoff of TM-score>0.7 to construct the structural profile. Out of the 87 test proteins, 41 have more than 10 structural analogs with TM-score>0.7. The average number of analogs is 51 for these proteins. In the remaining 46 cases, we gradually reduced the TM-score cutoff so that each target protein has at least 10 analogs to construct the structural profiles. As a result, 24 targets have the TM-score cutoff = 0.6 and 22 have TM-score cutoff = 0.5. In Figure 5, we present the I-TASSER folding results on the three groups of designed sequences, which indeed shows a difference in RMSD to the target structure. In general, when a higher number of closely analogous proteins are available, a better quality of structural profile can be constructed to closely characterize the conserved/mutation positions along the designed sequences. In our case, the average RMSD of the I-TASSER models for the first group of 46 proteins is 1.46 Å, which is lower than the two other groups based on the lower TM-score cutoffs (2.57 Å and 2.84 Å). On the contrary, the folding accuracy on the PBM sequences shows a reversed tendency, i.e. the RMSD of the third group with a TM-score cutoff >0.5 is lower than that of the first group with a TM-score cutoff >0.7; this is probably because of the slightly shorter chain length of the third group which is relatively easier to fold by I-TASSER.

In Figures S4 and S5, we also show the performance of torsion angles, secondary structure, solvation and sequence recapitulation on the different TM-score cutoffs. A similar dependence is observed on the torsion angles and secondary structures. But the TM-score cutoffs have no obvious influences on the solvation and sequence identities to the target scaffold.

These data may raise a concern with the design method for novel proteins which are supposed to have no close structural analogs in the PDB library. In reality, recent studies have demonstrated that the current PDB library is approaching to completeness and almost all the proteins, including random homopolypeptides, can have protein analogs with similar folds (TM-score>0.5) in the PDB [62]–[64]. To quantitatively examine this issue, we re-ran our design programs but excluded all analogous proteins with a TM-score>0.5 to the target. As a result, the average RMSD of the I-TASSER models indeed becomes relatively higher (increasing from 2.12 Å to 2.66 Å for the 87 test proteins) and the NRE for secondary structure, Φ/Ψ angles becomes larger (2.11, 0.6, 0.99, respectively). However, these are still much lower than the purely physics-based design methods, demonstrating that the structural profiles, even collected from distantly analogous proteins, are helpful for guiding the design procedures to construct better protein folds.

### Protein design based on the evolution terms only

Although the EBM design, combining both evolution and physics-based energy terms, demonstrated clear advantage over the PBM design that uses only the physics-based potentials, it is of interest to examine how the method works if the force field only includes the evolution-based terms (termed EvBM). In the third row of Table 1, we summarize the results when dropping off the FoldX terms from the EBM design, where the detailed data for each of the targets are listed in Table S3.

Overall, the EvBM results are largely comparable with the EBM method, but clearly outperform that by PBM, in terms of both the structural similarity of the I-TASSER models to the scaffolds and the quality of the local structural feature predictions. In summary, the TM-score of the I-TASSER models on the EvBM design is 11% higher than those created by PBM, and the normalized relative errors of SS, Φ, Ψ and SA by EvBM are 5-, 1.6-, 3.4- and 10-folds lower than that by PBM, respectively. The average sequence identity between design and scaffold along the entire chain by EvBM is comparable with that by EBM (27% vs. 28%), while that in the core region is the same as that by PBM (35%). These data demonstrate again that the evolution-based energy terms, including the profiles and the knowledge-based structure feature predictions, are the major driving force for the designs conducted by the EBM pipeline.

### A case study on *M. tuberculosis* proteins

Since the EBM design procedure is fully-automated, it has the potential for large-scale protein design applications. Here, as an illustration we apply the pipeline to redesign all solved proteins in *M. tuberculosis* (MTb) genome which contains various pathogens known to cause serious diseases in mammals, including tuberculosis and leprosy.

MTb proteins are encoded by 4,062 genes where 243 distinct proteins with length up to 296 residues have had their protein structure solved in the PDB library [65]. Table S5 summarizes the redesign results on all the 243 MTb proteins. Overall, the performance data is consistent with the results on the test proteins shown in Table 1. As shown in Table S5, the average NRE is 0.29, 0.14/0.18, and 0.09 for SS, Φ/Ψ, and SA, respectively. The average RMSD of the I-TASSER models is, however, relatively higher ( = 3.28 Å); this is mainly due to the difficulty of I-TASSER in folding large proteins since all homologous templates have been excluded from the template library. If we exclude the proteins of length >200 residues, the average RMSD of the I-TASSER models is reduced to 2.57 Å; but as expected, other qualities of local structural features (SS, Φ/Ψ, and SA) do not change much with the different length cutoffs.

In order to assess the diversity of the structural analogs used for profiling the MTb proteins, the number of the analogous structures and the average sequence identity of the analogs to the scaffolds are listed, respectively, in Columns 4 and 5 of Table S5. If we divide the results into different classes based on the number of structures needed for profile construction, then the trends follow the test set result as shown in Figure 5 and Figures S4, S5 (data not shown).

To partly examine the biological functionalities of the designed MTb proteins, we exploit a well-established structure-based ligand-binding prediction algorithm, COFACTOR [66]–[67], to search through the comprehensive ligand-protein interaction database, BioLiP [68], based on both local and global comparisons of I-TASSER models with template proteins. The analysis indicates that 62% of the EBM designed proteins have binding partners with a high confidence score, of which 51.3% are enzyme. When using the target scaffold structure as probe, COFACTOR detects slightly less (59%) binding partners, of which 50% are enzyme. Meanwhile, the EBM designed sequences have on average more binding sites (6.2 per protein) than the target proteins (5.5 per protein), although the number of binding residues per site are the same (7.2). In 88 out of the 117 cases (75%) where both designed and target sequences have the binding partner prediction with high confidence by COFACTOR on the same binding site, the binding affinity as assessed by the COFACTOR free-energy calculations is higher in the EBM proteins than in the target.

**Figure 6:**
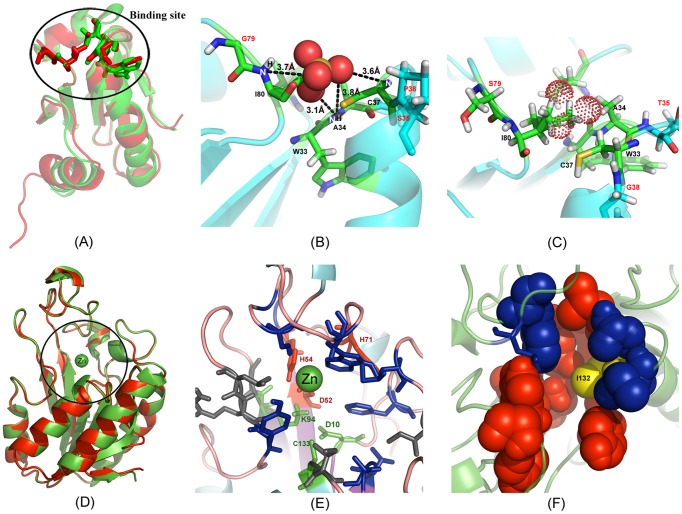
Illustrative examples of the EBM design on *M. tuberculosis* proteins. (A) Superposition of I-TASSER model of the EBM sequence (green) on the target structure from the thioredoxin C (red) with a RMSD 2.52 Å. (B) Sulfate ion binding with the designed protein where ion-protein hydrogen bonds are highlighted by dashed lines. (C) The EBM binding site analogous position on the target indicates that it cannot accommodate the sulfate ion (dotted sphere) due to steric overlaps. (D) Superposition of the PZAase protein (red) and the I-TASSER model on the EBM sequence (green) with a RMSD 0.28 Å. (E) Active site residues of PZAase as represented in sticks. Triad (green) and Zn^2+^ binding sites (red) are retained in the designed protein. Gray color indicates mutations at the active site. (F) Binding pockets identified by COFACTOR with red spacefill indicating an isochorismic acid binding site and blue the sulfate ion binding site. Y132I mutation in EBM design is designated by yellow. The figure was generated using Pymol and Adobe Photoshop software.

In Figures 6A–C, we show an illustrative example from the MTb thioredoxin C protein [PDB ID: 2I1U] where COFACTOR identifies a binding pocket with high confidence on the EBM protein, but no binding pocket is identified on the target. The designed sequence is 38% identical to the target protein where I-TASSER folds the sequence with an RMSD 2.52 Å of the first model to the target structure (Figure 6A). Interestingly, although no natural binding pocket exists in the target, mutations of T35S, G38P, and S79G on the designed sequence change the local binding pocket conformation and therefore facilitate the formation of four hydrogen bonds with the sulfate ion (dashed line in Figure 6B) that was identified by COFACTOR as the binding ligand. The binding affinity of this ion ligand is also favored by an independent binding scoring function, X-score [69], with an affinity score 3.45 pKd. As shown in Figure 6C, the ion-binding interaction vanishes in the target protein due to the dominant steric clashes of the side-chain atoms with the putative ligand.

In Figures 6D–F, we presented another example from the PZAase of Pyrococcus Horikoshii (PH999) which is known to bind with zinc [PDB ID: 1IM5]. The EBM design on PH999 shows a sequence identity 39% to the target and the I-TASSER model has an almost identical structure to the target (RMSD = 0.28 Å) (Figure 6D). Although we did not include the metal ion binding and active site information in the design procedure, the designed protein shows remarkable conservation within these regions. For instance, the triad consisting of C133, K94 and D10 (color green in Figure 6E), which occurs at the bottom of the cavity, and the residues (D52, H54, and H71) responsible for positioning zinc ion (Zn^2+^), are well preserved in the designed protein but the configuration of H71 is flipped in the model (red in Figure 6E). The cis-peptide bond observed in the target between V128 and A129 at the cavity of PH999 is also retained in the design. Among the 18 residues whose side-chains are involved at the active site, five have been mutated in the EBM design (V23I, A95G, E101L, A102R, and Y132I). All the mutations are spatially clustered together with other active site residues, except for Y132I where one aromatic hydrophilic residue is exchanged for a hydrophobic residue (yellow in Figure 6F). To examine the impact of the mutations and the aromatic exchange on overall ligand binding, we ran COFACTOR based on the I-TASSER model of the designed protein and the target structures, which identified one isochorismic acid binding site (Figure 6F; space filled atoms) including Residue-132 for both proteins with a binding affinity 5.1 pKd as calculated by X-Score. In addition, the COFACTOR predictions reveal one more sulfate ion binding site with a comparable binding affinity to the target protein (blue in Figure 6F). Apparently, the binding of isochorismic acid is highly competitive with that of sulfate ion in the target, both of which locate at nearly the same site. In the designed protein, the latter was completely eliminated, mainly due to the aromatic exchange at Y132I.

Despite the plausible analyses using state of the art computational docking scoring calculations, none of the binding data on the *M. Tuberculosis* proteins were experimentally validated, which is essential for the eventual confirmation of the biological insights. To facilitate further experimental studies, all designed sequences, the I-TASSER models, and the computational ligand-binding scoring analyses on *M. Tuberculosis* proteins are made available at: http://zhanglab.ccmb.med.umich.edu/MTb.

### Experimental validation of five EBM designed proteins

To experimentally validate the EBM designed sequences, we randomly selected five proteins: four from our benchmark set [heterogeneous nuclear ribonucleoprotein K domain (hnRNPK, PDBID: 1ZZK), thioredoxin domain (1R26), cytokine-independent survival kinase phox homology domain (CISK-PX, 1XTE), and light oxygen voltage domain (Lov2, 2V0U)], and one from the MTb genome [Translation Initiation Factor 1 (TIF1, 3I4O)]. These proteins contain different fold types (4 αβ- and 1 β-proteins) with length ranging from 68 to 146 residues. The RMSD of the I-TASSER models are in a typical range from 1.33 to 2.99 Å, with an average RMSD of 2.16 Å, close to the average RMSD of the overall benchmark test (2.12 Å). A list of the proteins is shown in Table 2.

**Table 2:** Summary of experimental validation results for the five designed sequences^a^.

Target	PDBID	Length	Type	RMSD^b^	Ep^c^	So^c^	SS^d^	3D^e^	α-helix%^f^	strand%^f^
hnRNPK	1ZZK	80	αβ	2.99 Å	+	+	+	−	32 (36)	16 (25)
thioredoxin	1R26	105	αβ	1.33 Å	+	+	+	+	36 (43)	21 (21)
CISK-PX	1XTE	116	αβ	2.06 Å	+	+	+	+	27 (39)	28 (28)
LOV2	2V0U	146	αβ	2.74 Å	+	+	+	−	31 (29)	24 (24)
TIF1	3I4O	68	β	1.67 Å	+	+	+	+	9 (9)	37 (54)

a The sign of “+” and “−” indicates positive and negative experimental results respectively.

b RMSD between the first I-TASSER model and the target scaffold.

c Protein expression and solubility determined by visual identification via comassie stain gels.

d Presence of secondary structural elements defined by circular dichroism.

e Possession of a stable tertiary fold determined by the presence of secondary structural elements (CD) and NMR spectroscopy.

f Percentage of α-helix residues decided by the CD spectra (the values in parentheses are the number in the scaffold structure.

For the designed sequences, constructs were first cloned into MSCG over-expression vectors with an N-terminal Mocr domain [70], expressed in a Rosetta 2 cell line (Millipore), purified to greater than 95% homogeneity, and then biophysicially characterized by circular dichroism and NMR spectroscopy. As seen in Table 2, all the designed domains successfully expressed and were soluble after the N-terminal Mocr tag was removed following purification.

**Figure 7:**
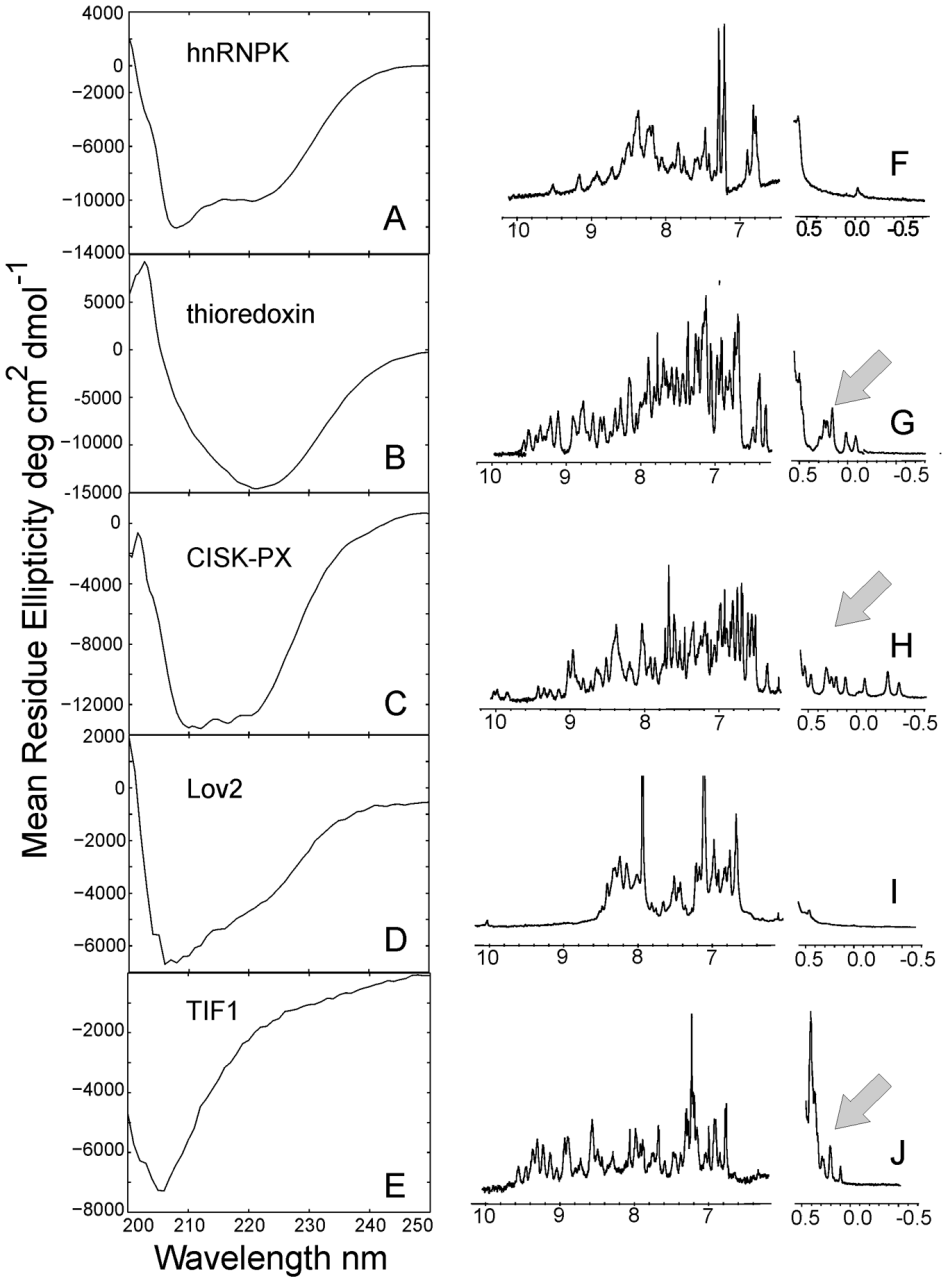
Circular dichroism and ^1^H 1D NMR spectrum of designed proteins. (A, F) hnRNPK; (B, G) thioredoxin; (C, H) CISK-PX; (D, I) Lov2; (E, J) TIF1 domains. The first column represents CD data, with X-axis representing the wavelength of circular polarized light (nm) and Y-axis the mean residue ellipticity measured in degree cm^2^ dmol^−1^. The second column consists of ^1^H 1D NMR spectra, with chemical shifts given in ppm on the X-axis. The arrows indicate key methyl chemical shifts indicated of a stable protein fold.

The domains were first biophysically characterized by circular dichroism to ascertain the presence of secondary structure. Again, all the designs had a negative ellipticity, as shown in the spectra from Figure 7 (Left Panel), indicating that these sequences possess distinct secondary structural elements. The hnRNPK and CISK-PX designs fit well to the typical α-helix/β-strand secondary structure folds with negative mean residue ellipticity troughs at 208 and 222 nm wavelength and clear exciton splitting. The Lov2 spectra have a major minimum at 208 nm plus a slop dip at 222 nm which also indicates a mixed α-helix/β-strand structure. The thioredoxin spectra have an unusual broad minimum at 222 nm, similar to the native *E. coli* thioredoxin spectra, suggests again a mixed helix/strand structure according to the analysis in [71]. In contrast, the TIF1 domain has almost no 222 nm signal and is dominated by β-strand spectra at 205 nm. In the last two columns of Table 2, we listed a quantitative comparison of fractions of α-helical/β-strand residues between the designed and the scaffold proteins, where the data on the designed proteins were calculated from an average of the estimations by three CD analysis programs (CONTINLL [72], CDSSTR [73], and SELCON [74]) on the CD spectra in Figure 7 and the secondary structures of the scaffolds were calculated by STRIDE [75]. The data showed that the secondary structure fractions in the designed sequences are largely consistent with those in the corresponding scaffold structures.

Following the CD experiments, the designed domains were analyzed by ^1^H 1D NMR spectroscopy. Specifically, we used NMR to probe the existence of a well-folded, stable, protein core, which is detectable by a shift of the side-chain resonances upfield (more negative) and by the dispersion and resolution of the amide protons. As shown in the Right Panel of Figure 7, the hnRNPK and Lov2 designs lacked upfield methyl chemical shifts (−1.0-0.5 ppm) and had sparse features in the protein amide range (5.5–10.0 ppm) (Figures 7F and 7I), which suggest that they do not possess a stable fold. By contrast, the designs for the thioredoxin, CISK-PX and TIF1 domains showed strongly shielded methyl shifts between 0.5 and −1.0 ppm and had well-resolved peaks in the amide region, features that are indicative of proteins possessing stable folds.

**Figure 8:**
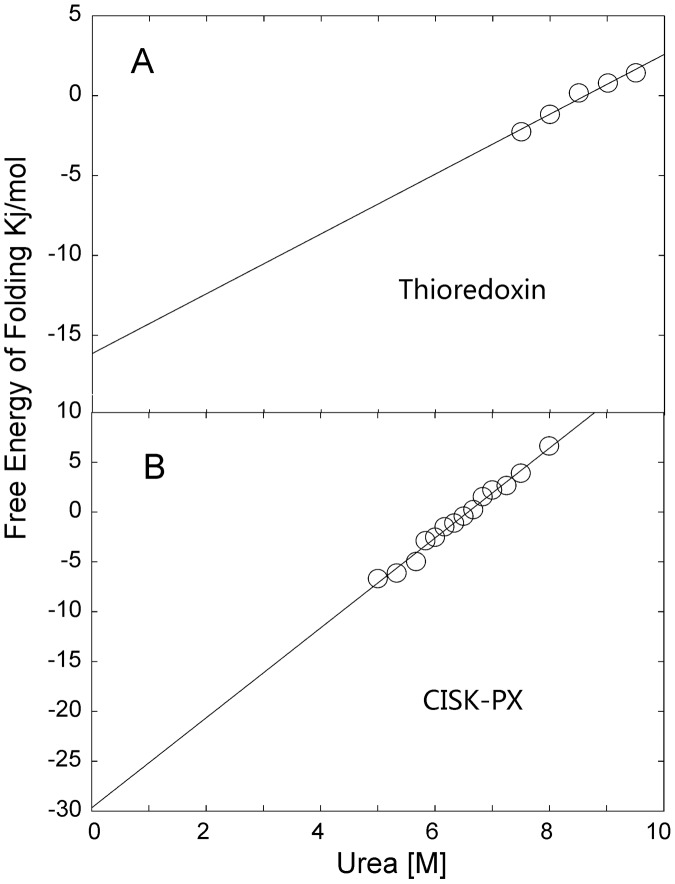
Free energy of folding was determined by circular dichroism. (A) thioredoxin; (B) CISK-PX domains. The figure plots free energy (kJ/mol) versus the concentration [M] of chemical denaturant urea. The unfolding assay was conducted in 25 mM NaPO_4_, 150 mM NaF, pH 7.5 with 2–3 uM protein concentration and 0–9.5 M urea concentrations at 298 K. The free energies of folding are equal to the intercept through linear regression [76].

Free energies of folding for the thioredoxin and CISK-PX designs were further determined by CD using urea as a chemical denaturant (we did not conduct the unfolding experiment on the TIF1 domain because it was observed to lack significant negative ellipticity at 222 nm). As shown in Figure 8, the designed thioredoxin domain started to unfold at ∼7.5 M urea and complete unfolding of the protein was not achieved with 9.5 M urea. In contrast, CISK-PX was completely unfolded by 8.5 M urea. Free energies of folding for the thioredoxin and CISK-PX domains were calculated by linear regression from the data points available and determined to be −16.1 and −29.6 kJ/mol, respectively [76]. Despite unfolding at a higher concentration of urea, the thioredoxin domain had a slower transition to an unfolded state and is thus less stable than the CISK-PX domain.

## Discussion

Deducing structure models from evolutionarily related proteins has been established to be the most reliable method for protein 3D structure prediction. Following a similar spirit, we introduce the idea of structure profile to protein design, a reverse procedure of protein folding and protein structure prediction. The key step of the approach is to construct an evolutionary profile from a family of proteins that have similar fold to the target structure. Such a profile matrix helps to identify the conserved/variable positions along the sequence, which are important in protein evolution and critical for maintaining the global structural fold and functionalities. Technically, since the foldable sequence space of a specific protein target is extremely narrow (few sequences can fold to the structure), targeting the design to an envelope of proteins of similar folds can increase the breadth of free-energy landscape and meanwhile enhance the robustness of the designed sequences upon structural variations.

One technical issue is that the profile-based design may simply converge to the consensus of the multiple sequence alignments, which can have discrete physiochemical feature distributions along the sequences. To alleviate the problem, we developed a set of single-sequence based predictors to regulate the secondary structure, torsion angles and solvent accessibilities. These predictors are fast and based only on a single sequence but the accuracy is comparable with the more sophisticatedly trained predictors using multiple sequence alignment searches (see Text S1 and Figures S1, S2, S3 for details). To accommodate the steric and physiochemical interactions of residues, a physics-based atomic potential (FoldX) is introduced on the top of the profile-based energy terms.

The method is tested on the design experiment of 87 non-homologous single-domain proteins covering different fold classes. Compared to the sequences designed only on the physics-based energy terms (PBM), significant improvement has been observed on the general features of the designed sequences, where the normalized relative errors are reduced by 7 times for secondary structure, 3 (5) times for Φ (Ψ) angles, and 21 times for solvent accessibilities. These improvements are partly attributed to the smoothening effect of the structural profile weighting and the restraints from the knowledge-based feature predictions. When submitting the sequences to the I-TASSER structure assembly pipeline, an average RMSD of 2.12 Å is achieved to the target structures although all homologous templates detectable by PSI-BLAST were excluded from our predictions (Table 1). Since the force field and phase space searching used by protein design and I-TASSER folding are independent from each other, such a high consistency between the I-TASSER models of the designed sequence and the target scaffold indicates that the design procedure using the structural profiling should have captured the features that are essential to generate the overall fold of the proteins.

The identity of the designed sequences to the target is relatively low (28%); but the identity of residues in the evolutionarily conserved regions is much higher (i.e. 44%; in comparison, only 23% residues are conserved in these regions in the PBM designed sequences). This data shows the effect of structural profiles in recapitulation of evolutionarily conserved positions. Meanwhile, the amino acid composition is closely similar to that of the target sequences. Compared to sequences generated by PBM, the absolute composition difference from the native sequence is reduced by more than three times (from 3.4% to 1.1%), and the bias of designed sequence to Proline is completely eliminated in EBM. The inclinations of hydrophobic residues over the hydrophilic and charged residues in PBM are also reduced greatly. These improvements facilitate the designed sequences in retaining the balance of the amino acid distributions along the sequence.

Because the design procedure is fully-automated, it has the potential for large-scale protein design applications. As an illustration, we applied the EBM method to redesign all 243 solved proteins from *M. tuberculosis*. The designed sequences can be folded by I-TASSER to the structures of average RMSD 3.28 Å without using homologous templates (or 2.57 Å for the proteins below 200 residues). In 75% of the cases where there is a confident binding partner on the same binding site, the binding affinity is higher in the EBM proteins than in the target, as shown by the binding free-energy calculated by COFACTOR [66] and X-score [69]. Two typical examples were shown in Figure 6: for thioredoxin, a new binding pocket was formed by the mutation of three key residues in the active site, which improved the binding pocket shape and hydrogen-bonding network with the ligand; for PZAase, although the overall sequence identity is only 39%, the triad, cis-peptide and metal ion binding residues at the active site are well conserved on the designed sequence. Nevertheless, one of the two competing binding sites (the one with sulfate ion) was eliminated by the mutation of Y132I in which one aromatic hydrophilic residue is replaced by a hydrophobic residue. Although these calculations were based only on computational docking analysis without stringent experimental screening, the converging data from different analysis methods show the possibility of varying substrate scope and binding affinity via the redesign of the active site residues to alter the catalytic activity of enzymes.

A handful of sequences were randomly selected from the EBM designed sequences for experimental validation. These sequences have a length ranging from 68 to 146 residues and cover different fold types. All the designed sequences were found to be soluble and possess distinct secondary structures as witnessed by the negative ellipticity in the circular dichroism experiment. Three out of the five sequences (thioredoxin, CISK-PX, and TIF1) were revealed to possessed stable tertiary structures by ^1^H 1D NMR spectra. Further, urea denaturation experiments combined with linear regression showed that the domains of thioredoxin and CISK-PX are stable with the free energies of folding below −16 kJ/mol. These experiments, although incomplete for all designed targets, demonstrated the EBM represents is a robust protein design tool capable of making novel sequences that adopt stable tertiary folds, with a quite reasonable success rate (∼3/5).

It should be mentioned that many methods in the literature have been developed to design proteins with either improved functions or completely novel folds through the mutation of natural sequences or *de novo* design calculations. One motivation for the development of EBM is to provide a *reliable* platform that can design any protein with improved foldability using the restraints from evolutionary profiles of similar fold families. With this platform, the functional characteristics, including enhanced and/or alternative ligand bindings for instance, can be further introduced. In a recent achievement (Brender et al, in preparation), we have demonstrated that the introduction of specific interface potentials to the current EBM platform be used to create altered binding affinity of natural or drug ligands on the designed proteins, as shown by computational scoring calculations as well as preliminary experimental data.

Overall, our study demonstrates the potential of using evolutionary based information in conjunction with the physics-based force field for *de novo* protein design. This opens up a new avenue in computational protein design to improve the biological and structural properties of the designed protein sequences. It also provides an exciting possibility to extend the existing template libraries for protein 3D structure predictions, which is under exploration in our lab.

## Materials and Methods

The evolution-based design procedure (EBM) consists of three steps: structural profile construction, Monte Carlo sequence space search, and final sequence selection (Figure 1).

### Structural profile construction

The first step of EBM is to construct a structure profile which will be used to guide the sequence design simulation and selection. For a given target protein structure (or scaffold), the profile is constructed from a family of structural analogs that are collected from a non-redundant set of the PDB library by the structural alignment program, TM-align [77], using the scaffold as the probe. For each structure alignment, the TM-align returns a TM-score to assesses the structural similarity of the PDB protein to the scaffold protein [78]. In general, the TM-score ranges from 0 to 1 with a higher value indicating a higher structural similarity, and a TM-score value>0.5 roughly corresponds to the similarity seen for proteins within the same SCOP/CATH family according to the database analyses [79]. In our design procedure, all PDB proteins with a TM-score>0.7 are considered to be a structure analog and added to the structural profile pool. If less than ten structural analogs are detected from the PDB with a TM-score>0.7, we gradually reduce the TM-score cutoff until the number of analogs is above ten to ensure a sufficient number of proteins for the followed-up profile construction.

To specify the conservation/variation residues in the analogy protein family, we construct a profile matrix following the idea of Gribskov et al [3], which was designed to extract the position-specific scoring table from the multiple sequence alignment (MSA). Here, the MSA is collected from the pair-wise TM-align structural alignments between the PDB protein and the scaffold but with the gaps/insertions eliminated according to the residues appearing along the scaffold sequence. The structural profile is specified by an *L×20* matrix, where *L* is the length of the scaffold sequence (and the MSA) and 20 is the number of different amino acid types. The elements of the matrix for amino acid *a* at position *p* is given by 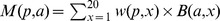. Here *B*(*a*, *x*) is the BLOSUM62 substitution matrix with *x* varying for 20 amino acids, and *w*(*p, x*) is the frequency of the amino acid *x* appearing at the *p*th position in the TM-align MSA. To account for the potential bias to specific protein families due to the uneven distribution of the PDB structures in the sequence space, we reweighted each residue in the MSA by a Henikoff-Henikoff scale *H*(*p*, *x*), i.e. 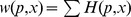.

Here, we note that the evolutionary profiles have often been defined from sequence-based homologous search, e.g. through hidden-Markov model [80] or PSI-BLAST [81] searches. The reason for us to choose structure analogs is due to the consideration that the profiles from structural analogs should be more sensitive to the desired folds of the scaffold, since numerous data analyses have shown that structure is more robust than sequence against the evolutionary variations and sequences of high residue identity may adopt completely different folds and functions [82]. Indeed, we have tried to use the sequence homologies instead of structural analogs or use the sequence homologies on top of the structural analogs for the profile construction, but found that the inclusion of sequence homologies increases the sequence diversity of the simulations, as well as increase the normalized relative errors and the RMSD of the I-TASSER predictions on the designed sequences.

### Monte Carlo sequence space search

Starting from a randomly generated sequence, Metropolis Monte Carlo simulations are conducted to search through the amino acid sequence space for the sequences that best match with the target structural profile. At each step of movement, a set of randomly selected residues will be mutated randomly.

The energy function of the MC search consists of two parts. The first part counts for the alignment match of the sequence decoy with the target structural profile:
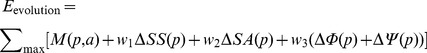
(1)where the first term is the structural profile defined above; the second, third and fourth terms in Eq. 1 are the difference of the decoy and target sequences in secondary structure, solvent accessibility and torsion angles, respectively. Because the predictions of these structural features are needed for each step of the MC movements, a quick neural-network predictor is developed based on single sequence for the decoy sequence which is much faster (takes ≪1 s) than the normal PSI-BLAST based predictors but with comparable prediction accuracy (see Text S1 and Figures S1, S2, S3 for details). The SS, SA and Φ/Ψ features for target structure is assigned by the DSSP program [56]. The optimal alignment path between the decoy and target structure is obtained by the Needleman-Wunsch dynamic programming with the maximum score assigned as *E*_evolution_ in Eq. 1.

The second part of the energy function contains a physics-based force field from FoldX V3.0b5 [53], designed to further enhance the stability and local structure packing of designed sequence. It consists of 9 empirical terms [53], [83]:
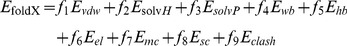
(2)where *E_vdw_* is the sum of the van der Waals contribution of all atoms; *E_solvH_* and *E_solvP_* count for the solvation energy for apolar and polar groups, respectively; *E_wb_* is the water bridge hydrogen bonding between water and protein; *E_hb_* is the intra-molecule hydrogen-bonding; *E_el_* counts for the electrostatic contribution of interactions between charged groups; *E_mc_* and *E_sc_* are entropy costs for fixing main-chain and side-chain atoms in a particular conformation, respectively; and *E_clash_* counts for the penalty from atomic steric overlaps. The parameters *f_1–9_* were trained by maximizing the correlation between the calculated and experimental free-energy changes on a set of experimental residue mutants. The detail of FoldX potential design and parameterization can be found in Refs [53], [83]. We used the default parameters for the FoldX calculation, except for the van der Waal weight *f_1_* which was increased to 0.33 to eliminate the extra steric clashes observed in our simulations. Since FoldX potential is full-atomic, we use SCWRL V4.0 [84] to construct the side-chain conformations after each MC movement.

To balance the two parts energy terms which are derived from different resources, we renormalized the energy terms based on their deviations:
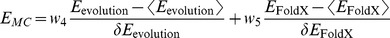
(3)where 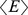 and δ*E* are average and standard deviation of the energy scores calculated from previous steps of simulations.

The weight parameters in Eqs 1 and 2 (*w_1–5_*) were determined on a set of 625 non-redundant training proteins that are non-homologous to the test set and case study proteins (see http://zhanglab.ccmb.med.umich.edu/EvoDesign/list625.txt). For *w_1–3_* in Eq. 1, the weights are decided by the relative accuracy of the individual feature predictions, i.e. w_1_ = C*A_SS_, w_2_ = C*A_SA_, w_3_ = C*A_TA_, where *A_SS_*, *A_SA_* and *A_TA_* are the number of correctly predicted residues on secondary structure (SS), solvent accessibility (SA) and torsion angles (TA), respectively, divided by the total number of residues on the training proteins. *C* is the parameter to balance the average magnitude of feature predictions with that of the profile term. The final weights for Eq. (1) are: *w_1_* = 1.58, *w_2_* = 2.45, *w_3_* = 1. For Eq. (2), the weights were adjusted so that the average contribution from the evolution terms and the physics based terms are comparable based on the designing simulation of the 625 training proteins. The final decided weights are *w_4_* = −0.5, *w_5_* = 1.22.

Following each of the random mutation trials, the movement is accepted or rejected by the Metropolis criterion, i.e. with the acceptance rate ∼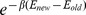, where *β* is the Boltzmann temperature factor and *E_new_* and *E_old_* are the energy calculated for the sequences after and before the mutation, respectively, based on Eq. (3).

We have conducted two control studies to the EBM design. In the first, the protein design by physics-based force field (PBM) was conducted following a procedure similar to the EBM simulation but the MC energy score contains only the FoldX function. i.e., we set *w_4_* = 0 and *w_5_* = 1 in Eq. (3). In the second, we consider only the evolution based energy potential, termed EvBM, i.e. we set *w_4_* = −1.0, *w_5_* = 0.0 in Eq. (3).

### Sequence clustering and designed sequence selection

For each target, ten independent Monte Carlo runs, each starting from a different random sequence, are performed. The final designed sequence is selected by clustering all the sequence decoys generated in Monte Carlo simulations. The clustering procedure is implemented by an algorithm similar to SPICKER with the distance matrix between sequence decoys defined by BLOSUM62 substitution scores, following the procedure by Bazzoli et al [19]. Initially, the distance threshold is set as zero but increases gradually until the size of the largest cluster reach to 40% of the total number of sequences. Upon termination, the sequence corresponding to the highest number of neighbors is considered a designed sequence.

In the Metropolis Monte Carlo simulations, the number of decoys at each sequence cluster *n_c_* is proportional to the partition function (*Z_c_*) of the conformational search, i.e. 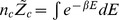, where the logarithm of the cluster size is thus related to the free-energy of the simulation by 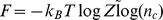. Thus, the design sequences with the largest cluster size in EBM should correspond to the state of the lowest free-energy in our simulations.

### Computation time of current method and possible improvement on kinetics

Our sequence design simulations contain the calculation of two parts of energy terms, Eqs. (1) and (2). Since the structure feature prediction is single-sequence dependent, the calculation of the evolutionary terms is fast and takes only fraction of seconds per sequence per Monte Carlo step. The calculation of the second term from FoldX is however much more expensive (up to minutes per sequence) which is mainly due to the side-chain conformation calculation by the SCWRL program. In our test on the 87 proteins, the average simulation time of our method without using FoldX is 3.8 hours, while including FoldX increases the time for the program to 16.5 hours.

Another important factor impacting the time cost and the quality of designed sequences is the dynamics of the Monte Carlo simulations. The current EBM programs implemented 10 independent Metropolis MC runs, each running 30,000 mutation movements. Due to the inherent limit of the Metropolis algorithm whereby the acceptance rate of movements is proportional to the inversed exponential of the height of energy barriers (see above), the individual MC simulations can be easily trapped at local minimum. In our test, the pair-wise sequence identity between the lowest energy sequences of ten different runs is low (28% on average), which demonstrated the divergence of the Metropolis simulations and some simulations might have trapped at local minimum. Accordingly, the average RMSD of the I-TASSER models on the ten lowest energy sequences from the ten MC runs is 3.7 Å, 1.5 Å higher than that obtained for sequences selected from clustering (see Table 1), which demonstrates the necessity of multiple MC runs and the usefulness of sequence clustering.

We also tested the simulations starting from one of the sequences used in the profile or from the consensus of the MSA. The average results are similar to that of the simulations starting from random sequences, including the average RMSD of the I-TASSER models of the designed sequences and the pair-wise sequence identity between the lowest energy sequences of different runs. The average identity between final sequence and the starting seeds is also low (<30%), which indicates that the simulations did not stick to the seeds when starting from the sequences in the profile. The similar average results were also obtained if we start the 10 independent simulations from the same sequence but with different random numbers. Nevertheless, we choose to have the EBM simulations started from different random sequences in our designs, which should help avoid the possible bias as introduced by specific starting sequences.

Finally, we have tested running more than 10 MC simulations, but the assessment results are not improved compared to the 10 runs, in terms of average sequence identity, normalized relative error and the RMSD of the final I-TASSER models to scaffold. The data implies that 10 simulation runs are probably sufficient for obtaining converged results. Nevertheless, 10 runs might still be too expensive, especially for large-scale design applications. We are testing the use of more advanced MC techniques, including replica-exchange sampling [85] and simulated annealing [86], with the aim to improving the speed and kinetics of the EBM simulations. The results will be reported elsewhere.

### Expression constructs

Five proteins were randomly selected from the EBM based design for experimental validation. The DNA and protein sequences of the proteins are listed in Text S1. The DNA sequences for the designed domains were cloned into a mOCR domain over-expression vector via ligation independent cloning [70]. The expression construct contains a N-terminal solubility tag consisting of 6×His tag, a Mocr solubility domain, and a rTEV protease site. The following N-terminal artificial cloning residues, “SNA”, remain after rTEV protease cleavage during purification.

### Protein expression and purification

Design constructs were transformed into a Rosetta 2 E. coli expression cell strain (EMDmillipore). Cells were grown in LB media with ampicillin at 0.1 g/L at 310 K until mid-log phase. At a cell density of 0.6–1.0 OD (600 nm wavelength) protein expression was induced by the addition of 0.2 mm IPTG for 5 hours at 305 K. All temperatures were at 277 K during purification and biophysical characterization unless declared otherwise. Cells were harvested by centrifugation and resuspended in 50 mM Tris pH 7.5, 150 mM NaCl, 5 mM imidazole, and then lysed by sonication (Fisher model 705 series). Samples were subsequently centrifuged [30,000 g×30 min Beckman J26-XP (JA25.50 rotor)] to pellet cell debris. The supernatant was incubated with Ni-NTA resin (Qiagen) and washed with 50 bed volumes of resuspension buffer. The protein was subsequently eluted with resuspension buffer plus 200 mM imidazole. After being dialyzed overnight in resuspension buffer and rTEV protease, the cleaved N-terminal tag containing the mOCR domain was removed via substractive Ni-NTA and anion exchange Acro-sep Q (Pall) purification. The eluate was subsequently concentrated using 3–10 K MWCO concentrators (Pall). A final purification polishing step by size-exclusion gel filtration using a (GE) AKTA chromatographic work station and an S-100 column was conducted.

### Biophysical characterization

Circular dichroism spectroscopy was conducted to determine if the designed domains had secondary structure. An Aviv 202 CD spectropolarimeter was used for all experiments. Wavelength scans from 190–250 nm were conducted with 2 sec averaging and 1.5 nm slit width. Experimental conditions were 20 mM NaPO_4_ pH 7.5, 50 mM NaCl at 288 K. Protein concentration was 2–4 µM. Measurements were in millidegrees ellipticity, which was then converted to mean residue ellipticity (MRE) for analysis. Data was collected in triplicate, and averaged. To analyze protein stability, unfolding experiments were conducted in 25 mM NaPO_4_ pH 7.5, 150 mM NaF at 298 K with increasing concentrations of urea as a denaturant (up to 9.5 M). Ellipticity values at 222 nm were recorded for each sample.

NMR spectroscopy was conducted to determine if the designed domains possessed stable tertiary folds. ^1^H 1D NMR spectra were recorded using a Bruker 600 MHZ spectrometer with cryoprobe at 20 mM NaPO_4_ pH 7.5, 150 mM NaCl, and 298 K with protein concentrations ranging from 70–100 µM.

## Supporting Information

Figure S1 Illustration of amino acid frequency counting within a window size of ±three residues.
(TIF)

Figure S2 Illustration of fingerprint assignments from neural network SS training where SS propensity score, amino acid composition score, and BLOSUM62 substitution matrix are listed side-by-side with a separation of a noise (black filled cell).
(TIF)

Figure S3 Illustration of fingerprint assignments from SA neural network training where SA propensity score, secondary structure prediction, amino acid composition score, and BLOSUM62 substitution score are listed side-by-side with a separation of a noise (black filled cell).
(TIF)

Figure S4 The average normalized relative error (NRE) of the structural features of the designed sequence relative to the DSSP assignments. (A) Backbone torsion angles (Φ/Ψ); (B) Secondary structure (SS); (C) Solvent accessibility (SA). Along the X-axis, the dataset is divided based on the TM-score cutoff on the templates that were used for constructing sequence profiles.
(TIF)

Figure S5 Average sequence identity of the designed sequences to the target sequences. ‘All’ indicates overall sequence identity and ‘core’ indicates the identity at the core of the proteins. Along with X-axis, the dataset is divided based on TM-score cutoff on the template proteins that are used for constructing the sequence profiles.
(TIF)

Table S1 List of designed proteins from previous experiments.
(PDF)

Table S2 Summary of EBM design on the 87 test proteins.
(PDF)

Table S3 Summary of EvBM design on the 87 test proteins.
(PDF)

Table S4 Summary of PBM design on the 87 test proteins.
(PDF)

Table S5 Summary of EBM based protein design on the *M. tuberculosis* genome. The data has been sorted by the normalized relative error (NRE) on secondary structure.
(PDF)

Text S1 Structure feature predictions and DNA/protein sequences.
(PDF)
