# Supplementary figures and images for: An Evolution-Based Approach to De Novo Protein Design and Case Study on Mycobacterium tuberculosis
Source: PLoS Comput Biol. 2013 Oct 24;9(10):e1003298. doi: 10.1371/journal.pcbi.1003298 (PMC3812052; doi:10.1371/journal.pcbi.1003298)

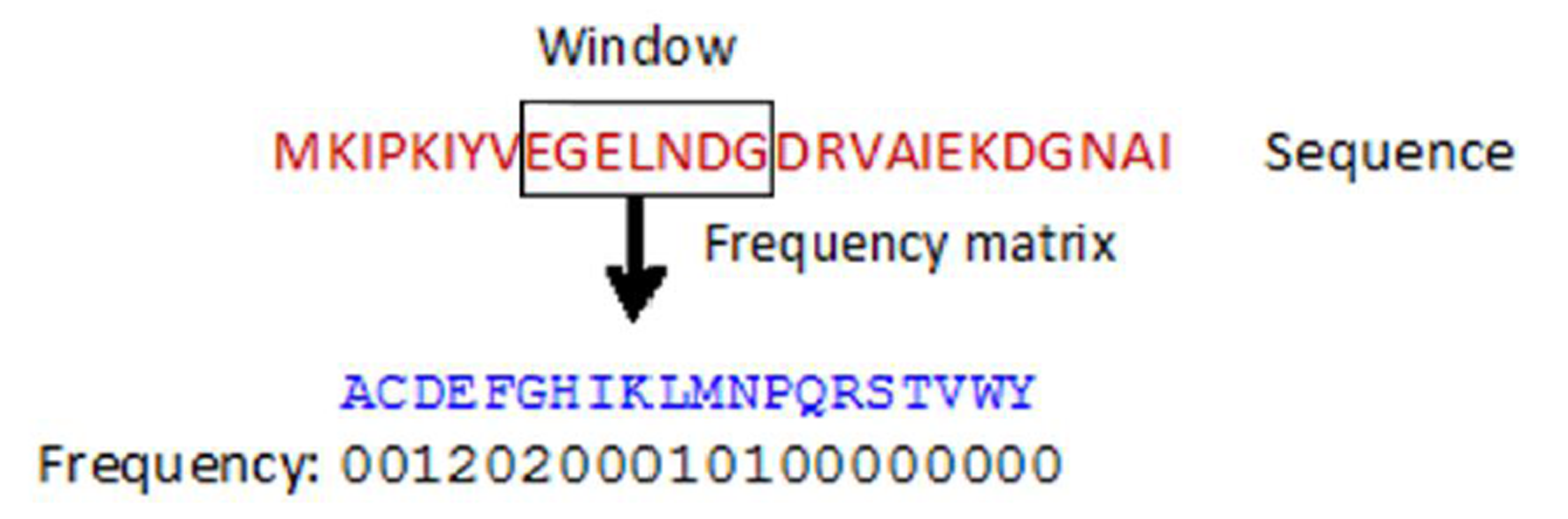

Supplement: Figure S1 — Illustration of amino acid frequency counting within a window size of ±three residues. (TIF) [file pcbi.1003298.s001.tif]

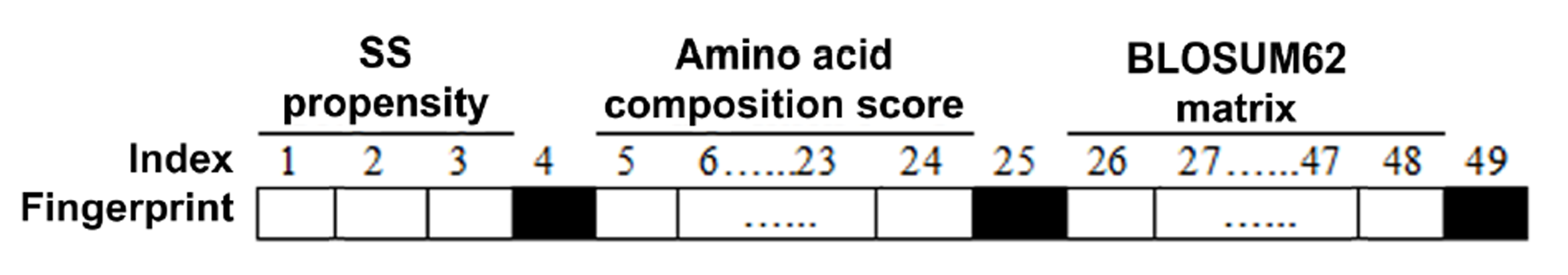

Supplement: Figure S2 — Illustration of fingerprint assignments from neural network SS training where SS propensity score, amino acid composition score, and BLOSUM62 substitution matrix are listed side-by-side with a separation of a noise (black filled cell). (TIF) [file pcbi.1003298.s002.tif]

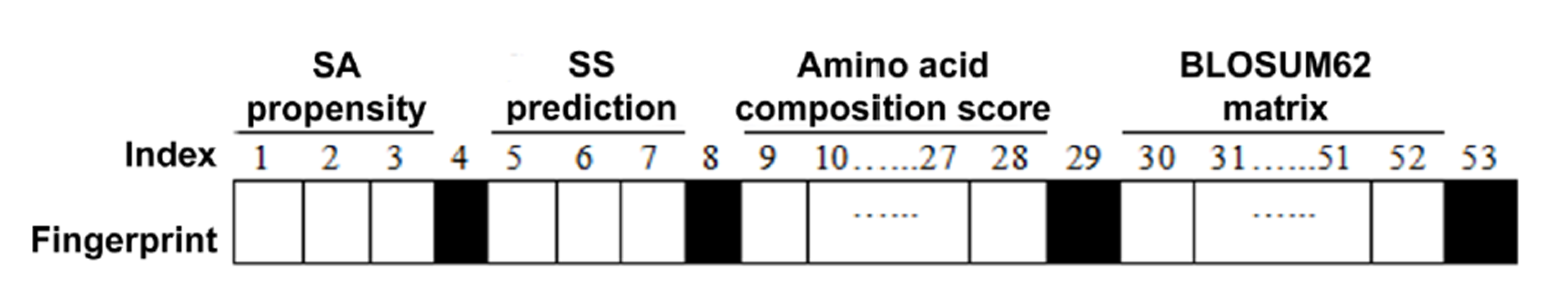

Supplement: Figure S3 — Illustration of fingerprint assignments from SA neural network training where SA propensity score, secondary structure prediction, amino acid composition score, and BLOSUM62 substitution score are listed side-by-side with a separation of a noise (black filled cell). (TIF) [file pcbi.1003298.s003.tif]

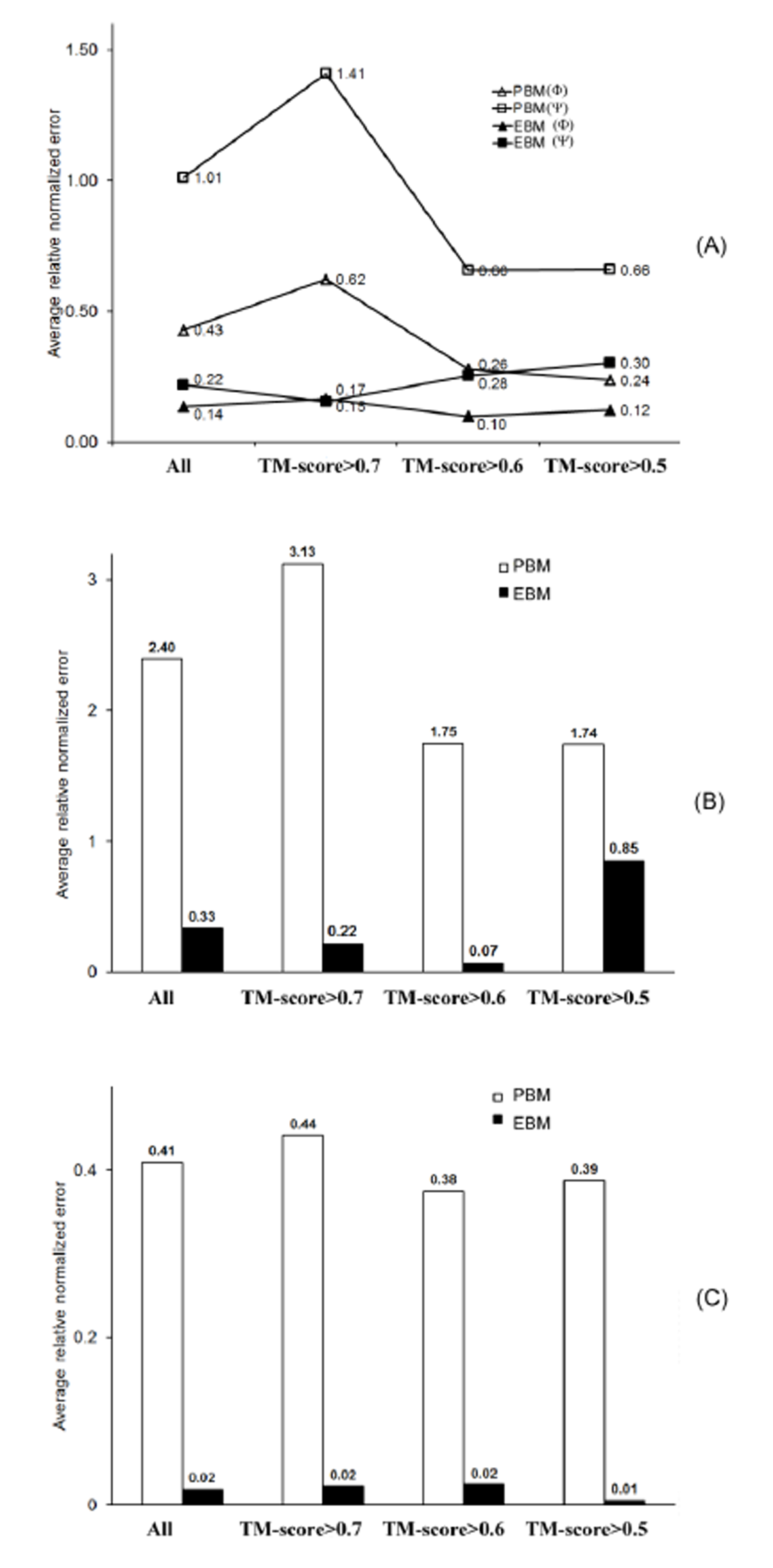

Supplement: Figure S4 — The average normalized relative error (NRE) of the structural features of the designed sequence relative to the DSSP assignments. (A) Backbone torsion angles (Φ/Ψ); (B) Secondary structure (SS); (C) Solvent accessibility (SA). Along the X-axis, the dataset is divided based on the TM-score cutoff on the templates that were used for constructing sequence profiles. (TIF) [file pcbi.1003298.s004.tif]

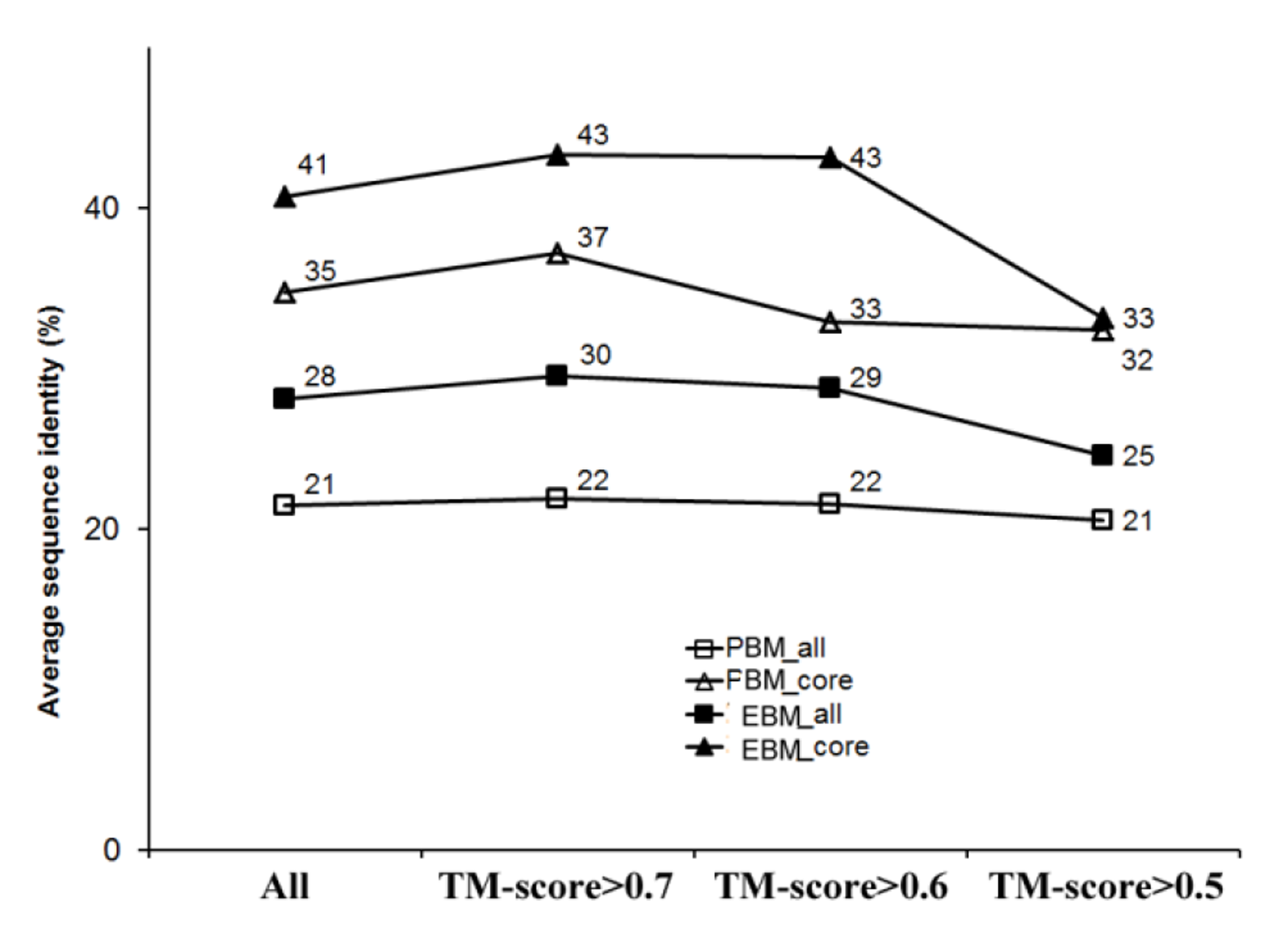

Supplement: Figure S5 — Average sequence identity of the designed sequences to the target sequences. ‘All’ indicates overall sequence identity and ‘core’ indicates the identity at the core of the proteins. Along with X-axis, the dataset is divided based on TM-score cutoff on the template proteins that are used for constructing the sequence profiles. (TIF) [file pcbi.1003298.s005.tif]
