# Supplementary material for: An Evolution-Based Approach to De Novo Protein Design and Case Study on Mycobacterium tuberculosis
Source: PLoS Comput Biol. 2013 Oct 24;9(10):e1003298. doi: 10.1371/journal.pcbi.1003298 (PMC3812052; doi:10.1371/journal.pcbi.1003298)
Supplement: Table S1 — List of designed proteins from previous experiments. (PDF) [file pcbi.1003298.s006.pdf]

Table S1. List of designed proteins from previous experiments.

Sequence ID	PDB ID	Length	Failed step[a]	References
1lq7A	1lq7A	67	Success	Dai et al [1]
1mj0A	1mj0A	156	Success	Kohl et al [2]
1p68A	1p68A	102	Success	Wei et al [3]
1qysA	1qysA	91	Success	Kuhlman et al [4]
1vjqA	1vjqA	71	Success	Dantas et al [5]
2a3dA	2a3dA	73	Success	Walsh et al [6]
2cw1A	2cw1A	65	Success	Walsh et al [6]
2juaA	2juaA	102	Success	Go et al [7]
2jvfA	2jvfA	94	Success	Stordeur et al [8]
2kl8A	2kl8A	85	Success	Koga et al [9]
2ln3A	2ln3A	83	Success	Koga et al [9]
2ltaA	2ltaA	110	Success	Koga et al [9]
2lv8A	2lv8A	110	Success	Koga et al [9]
2lvbA	2lvbA	112	Success	Koga et al [9]
2qyjA	2qyjA	154	Success	Merz et al [10]
3b83A	3b83A	95	Success	Hu et al [11]
MSP2-FC27	/	237	Natively unfolded	Zhang et al [12]
alpha-synuclein	/	140	Natively unfolded	Eliezer et al [13]
4EbindingproteinI	/	118	Natively unfolded	Fletcher et al [14]
4EbindingproteinII	/	120	Natively unfolded	Fletcher et al [14]
Fold-I-11	/	76	Not soluble	Koga et al [9]
Fold-I-2	/	76	Not folded-CD	Koga et al [9]
Fold-I-4	/	76	Not folded-CD	Koga et al [9]
Fold-I-8	/	76	Not folded NMR	Koga et al [9]
Fold-II-8	/	99	Not folded-CD	Koga et al [9]
Fold-II-9	/	99	Not folded-CD	Koga et al [9]
Fold-II-1	/	99	Not folded NMR	Koga et al [9]
Fold-II-3	/	99	Not folded NMR	Koga et al [9]
Fold-II-11	/	99	Not folded NMR	Koga et al [9]
Fold-III-6	/	72	Not soluble	Koga et al [9]
Fold-III-7	/	72	Not soluble	Koga et al [9]
Fold-III-1	/	72	Incorrect CD	Koga et al [9]
Fold-III-13	/	72	Not folded-CD	Koga et al [9]
Fold-III-4	/	72	Not folded NMR	Koga et al [9]
Fold-IV-2	/	101	Not folded NMR	Koga et al [9]
Fold-V-8	/	99	Not soluble	Koga et al [9]
Fold-V-1	/	99	Not folded-CD	Koga et al [9]
Fold-V-2	/	99	Not folded-CD	Koga et al [9]
Fold-V-4	/	99	Not folded-CD	Koga et al [9]
Fold-V-9	/	99	Not folded-CD	Koga et al [9]
Fold-V-10	/	99	Not folded-CD	Koga et al [9]
Fold-V-11	/	99	Not folded-CD	Koga et al [9]

Fold-V-12	/	99	Not folded-CD	Koga et al [9]
MDM2a	/	85	Not folded-CD	Shultis et al [15]
MDM2b	/	85	Not folded-CD	Shultis et al [15]

[a]**Success**: protein structure was solvable by NMR or X-ray crystallography; **Natively unfolded**: biologically expressed (i.e. not designed) protein experimentally known to be unfolded in the native state; **Not soluble**: protein was not soluble after expression (usually indicative of improperly exposed hydrophobic surfaces); **Not folded-CD**: largely random coil secondary structure by CD; **Incorrect CD**: secondary structure by CD not consistent with predicted structure; **Not folded NMR**: poorly resolved NMR spectra reflective of a dynamic (unstable) protein core.

References

1. Dai QH, Tommos C, Fuentes EJ, Blomberg MR, Dutton PL, et al. (2002) Structure of a de novo designed protein model of radical enzymes. J Am Chem Soc 124: 10952-10953.
2. Kohl A, Binz HK, Forrer P, Stumpp MT, Pluckthun A, et al. (2003) Designed to be stable: crystal structure of a consensus ankyrin repeat protein. Proc Natl Acad Sci U S A 100: 1700-1705.
3. Wei Y, Kim S, Fela D, Baum J, Hecht MH (2003) Solution structure of a de novo protein from a designed combinatorial library. Proc Natl Acad Sci U S A 100: 13270-13273.
4. Kuhlman B, Dantas G, Ireton GC, Varani G, Stoddard BL, et al. (2003) Design of a novel globular protein fold with atomic-level accuracy. Science 302: 1364-1368.
5. Dantas G, Corrent C, Reichow SL, Havranek JJ, Eletr ZM, et al. (2007) High-resolution structural and thermodynamic analysis of extreme stabilization of human procarboxypeptidase by computational protein design. Journal of Molecular Biology 366: 1209-1221.
6. Walsh ST, Cheng H, Bryson JW, Roder H, DeGrado WF (1999) Solution structure and dynamics of a de novo designed three-helix bundle protein. Proc Natl Acad Sci U S A 96: 5486-5491.
7. Go A, Kim S, Baum J, Hecht MH (2008) Structure and dynamics of de novo proteins from a designed superfamily of 4-helix bundles. Protein Sci 17: 821-832.
8. Stordeur C, Dalluge R, Birkenmeier O, Wienk H, Rudolph R, et al. (2008) The NMR solution structure of the artificial protein M7 matches the computationally designed model. Proteins 72: 1104-1107.
9. Koga N, Tatsumi-Koga R, Liu G, Xiao R, Acton TB, et al. (2012) Principles for designing ideal protein structures. Nature 491: 222-227.
10. Merz T, Wetzel SK, Firbank S, Pluckthun A, Grutter MG, et al. (2008) Stabilizing ionic interactions in a full-consensus ankyrin repeat protein. Journal of Molecular Biology 376: 232-240.
11. Hu X, Wang H, Ke H, Kuhlman B (2008) Computer-based redesign of a beta sandwich protein suggests that extensive negative design is not required for de novo beta sheet design. Structure 16: 1799-1805.

12. Zhang X, Perugini MA, Yao S, Adda CG, Murphy VJ, et al. (2008) Solution conformation, backbone dynamics and lipid interactions of the intrinsically unstructured malaria surface protein MSP2. Journal of Molecular Biology 379: 105-121.
13. Eliezer D, Kutluay E, Bussell R, Jr., Browne G (2001) Conformational properties of alpha-synuclein in its free and lipid-associated states. Journal of Molecular Biology 307: 1061-1073.
14. Fletcher CM, McGuire AM, Gingras AC, Li H, Matsuo H, et al. (1998) 4E binding proteins inhibit the translation factor eIF4E without folded structure. Biochemistry 37: 9-15.
15. Shultis D, Mitra P, Zhang Y (2013) Unpublished results.
