# Supplementary material for: An Evolution-Based Approach to De Novo Protein Design and Case Study on Mycobacterium tuberculosis
Source: PLoS Comput Biol. 2013 Oct 24;9(10):e1003298. doi: 10.1371/journal.pcbi.1003298 (PMC3812052; doi:10.1371/journal.pcbi.1003298)
Supplement: Table S2 — Summary of EBM design on the 87 test proteins. (PDF) [file pcbi.1003298.s007.pdf]

Table S2. Summary of EBM design on the 87 test proteins.

PDBID	L[a]	SC[b]	aTM[c]	TM[d]	R (Å)[e]	Normalized relative error				Sequence identity (%)	
						SS	Φ	Ψ	SA	All	Core
1A2P_A	109	a+b	0.36	0.99	0.37	0.06	0.17	0.08	-0.05	42	44
1ABA_A	88	a/b	0.66	0.87	1.93	0.20	0.27	0.17	-0.01	34	54
1BKR_A	109	a	0.82	0.99	0.29	-0.22	0.02	-0.11	-0.02	43	63
1DBW_A	124	a/b	0.76	0.88	1.71	0.00	-0.03	-0.05	-0.08	28	38
1EAQ_A	125	b	0.64	0.91	1.45	-0.10	-0.06	-0.20	-0.13	30	54
1EW4_A	107	a+b	0.50	0.95	0.92	0.92	0.24	0.42	0.05	17	21
1F46_A	140	a+b	0.55	0.98	0.45	-0.09	0.01	0.09	-0.05	28	42
1GBS_A	186	a+b	0.67	0.99	0.18	1.30	0.02	-0.04	0.04	46	53
1GUT_A	52	b	0.74	0.97	0.36	0.33	0.09	-0.05	0.08	25	44
1HZT_A	151	a+b	0.77	0.99	0.45	-0.13	0.43	0.11	0.04	25	33
1I2T_A	62	a	0.59	0.96	0.51	0.14	-0.20	0.58	0.13	20	50
1IDP_A	148	a+b	0.79	0.94	1.20	0.03	-0.06	-0.01	-0.02	24	30
1IUJ_A	98	a+b	0.78	0.78	2.22	0.71	-0.07	-0.13	-0.17	25	36
1JB3_A	128	b	0.60	0.98	0.51	-0.04	0.23	0.26	0.03	25	37
1JF8_A	131	a/b	0.90	0.99	0.44	-0.36	0.03	0.44	0.06	35	50
1KMT_A	139	b	0.66	0.99	0.31	0.07	0.09	0.21	-0.04	27	30
1KNG_A	145	a/b	0.76	0.96	0.88	0.19	0.06	0.18	0.01	30	39
1KQ1_A	61	b	0.81	0.96	0.50	0.29	0.16	0.25	0.01	28	62
1M9Z_A	106	small	0.60	0.96	0.83	0.13	0.11	0.06	-0.01	35	59
1MF7_A	195	a/b	0.83	0.95	1.36	0.08	0.26	0.03	0.02	27	36
1MG4_A	102	a+b	0.50	0.63	3.55	1.83	0.50	0.48	0.24	14	23
1NXM_A	195	b	0.56	0.96	1.13	0.27	0.21	0.27	0.00	23	30
1NZ0_A	110	a+b	0.52	0.84	1.75	0.53	0.29	0.53	0.10	14	13
1O7I_A	116	b	0.81	0.94	1.01	0.00	0.20	-0.10	0.00	30	41
1OAI_A	58	a	0.67	0.85	1.48	-0.80	0.60	1.73	0.04	28	31
1OH0_A	126	a+b	0.78	0.95	1.17	-0.05	0.14	0.10	0.00	27	32
1OK0_A	75	b	0.62	0.60	7.22	0.00	0.00	-0.09	0.07	18	45
1QHQ_A	140	b	0.65	0.37	11.00	0.24	0.17	0.06	0.20	35	38
1R26_A	106	a/b	0.79	0.93	1.01	0.20	0.15	-0.08	0.01	34	51
1R6J_A	83	b	0.82	0.89	1.68	0.00	0.39	0.28	0.03	24	40
1SHU_X	182	a/b	0.84	0.91	1.73	0.25	0.25	0.10	0.06	23	38
1T3Y_A	132	a+b	0.88	0.85	1.97	0.00	-0.03	-0.12	0.06	19	33
1TQG_A	106	a	0.75	0.97	0.64	1.50	0.22	1.01	-0.02	19	33
1TUK_A	68	a	0.53	0.38	5.30	0.33	0.12	0.98	0.06	9	11
1UCS_A	65	b	0.75	0.97	0.50	-0.05	-0.13	-0.12	0.05	53	67
1URR_A	98	a+b	0.85	0.95	0.80	0.13	0.08	0.16	-0.01	34	50
1UTG_A	71	a	0.50	0.98	0.42	2.33	-0.25	0.30	-0.19	10	10
1V5I_B	71	a+b	0.65	0.87	1.36	-0.43	0.10	0.77	0.02	24	45
1VH5_A	139	a+b	0.81	0.98	0.64	0.88	0.45	0.27	0.03	25	33
1VKK_A	138	a+b	0.86	0.91	1.70	0.06	0.19	0.38	0.00	29	32
1VQS_A	105	a+b	0.66	0.98	0.56	0.05	0.49	0.28	0.03	15	21

1VZI_A	126	b	0.32	0.18	16.93	1.93	0.36	0.79	0.30	17	33
1WLU_A	118	a+b	0.79	0.98	0.45	0.14	0.33	0.10	0.00	29	26
1X6Z_A	120	a+b	0.70	0.93	1.20	-0.24	0.19	0.13	-0.02	34	43
1XTE_A	117	a+b	0.81	0.97	0.71	0.00	0.27	0.21	-0.05	32	56
1ZHV_A	129	a+b	0.80	0.57	8.26	1.08	0.26	1.14	0.18	18	21
1ZKE_A	82	a	0.61	0.92	1.11	-0.22	-0.16	-0.47	0.05	14	18
1ZZK_A	81	a+b	0.79	0.89	2.99	-0.29	0.57	0.19	0.00	36	50
2ANX_A	146	a+b	0.81	0.99	0.30	0.04	0.18	0.04	0.06	30	34
2BWF_A	76	a+b	0.81	0.90	1.31	-0.35	0.34	0.15	0.00	32	65
2C9Q_A	103	b	0.63	0.86	1.83	0.22	-0.18	0.17	0.02	30	36
2CAR_A	197	a/b	0.78	0.99	0.44	-0.06	0.05	0.04	-0.49	29	36
2CMP_A	53	a	0.52	0.94	0.61	3.00	0.00	0.63	0.21	25	46
2CVI_A	78	a+b	0.76	0.89	1.17	0.08	0.14	0.21	-0.04	30	41
2D3D_A	84	a	0.66	0.91	1.15	-0.16	0.22	0.39	-0.05	31	54
2ERB_A	124	a	0.78	0.99	0.37	0.33	0.09	0.09	0.01	29	47
2F01_A	122	b	0.70	0.98	0.76	0.04	-0.07	-0.18	-0.09	39	59
2FTR_A	97	a+b	0.79	0.90	1.36	0.00	0.01	0.00	-0.06	26	36
2GMY_A	148	a	0.64	0.99	0.29	0.42	0.33	0.45	0.02	20	30
2GPI_A	92	a+b	0.49	0.82	1.92	0.43	0.19	0.11	0.07	9	17
2J2J_A	183	b	0.90	0.77	3.62	-0.35	0.23	0.14	0.10	23	36
2J5Y_A	55	a	0.77	0.91	1.24	4.00	-0.39	-0.04	-0.18	35	55
2J8B_A	79	small	0.70	0.90	1.16	0.00	0.04	0.15	-0.03	32	52
2O1Q_A	140	b	0.68	0.53	10.01	1.08	0.35	0.42	0.22	17	26
2O9S_A	68	b	0.76	0.83	3.15	0.12	0.08	0.43	0.10	39	74
2P5K_A	64	a	0.64	0.96	0.53	0.00	0.02	0.52	-0.01	38	63
2PR7_A	138	a/b	0.78	0.70	4.83	0.25	0.22	0.22	0.25	27	39
2PTH_A	194	a/b	0.71	0.98	0.58	0.00	0.18	0.01	0.00	25	37
2PV2_A	104	a+b	0.91	0.96	0.69	0.36	0.15	-0.11	0.03	32	59
2QCP_X	77	b	0.77	0.94	0.77	0.83	1.14	0.95	0.30	18	42
2V0U_A	147	a/b	0.77	0.92	2.65	0.15	0.02	0.12	-0.03	54	70
2V1Q_A	60	b	0.82	0.91	1.01	0.00	0.22	0.48	0.12	34	36
2VMH_A	148	b	0.71	0.97	0.95	0.18	-0.03	0.22	0.05	24	25
2VPB_A	58	a+b	0.68	0.86	1.15	-0.07	-0.15	-0.13	-0.03	39	64
2VZC_A	128	a	0.79	0.93	1.24	0.29	0.26	0.60	0.18	19	34
2WLV_A	145	a/b	0.75	0.92	1.49	0.05	0.05	0.19	-0.02	33	42
2ZXY_A	87	a	0.63	0.69	4.22	-0.18	0.09	0.02	0.01	36	55
3CTG_A	109	a/b	0.89	0.97	0.76	-0.25	0.05	0.03	0.00	39	54
3E9T_A	113	b	0.86	0.99	0.31	0.11	-0.01	0.03	0.01	39	41
3EBT_A	132	a+b	0.78	0.92	1.47	-0.11	-0.05	0.01	-0.01	29	33
3EF8_A	150	a+b	0.74	0.80	8.05	0.52	0.39	0.29	-0.02	23	27
3FEA_A	84	a+b	0.57	0.97	0.57	-0.06	0.18	-0.06	-0.02	42	69
3FIL_A	57	a+b	0.84	0.92	0.73	-0.11	-0.20	-0.20	0.01	63	92
3G21_A	78	a	0.45	0.36	11.57	3.57	0.12	1.26	0.18	12	16
3G36_A	53	a	0.43	0.32	11.19	1.67	-0.23	-0.36	-0.45	21	0
3IV4_A	107	a/b	0.80	0.82	1.95	-0.36	-0.07	-0.07	0.04	22	34
3VUB_A	102	b	0.70	0.88	1.54	0.28	0.12	0.33	0.06	18	31
Average	111		0.71	0.87	2.12	0.33	0.14	0.22	0.02	28	41

[a]Length of targets.
[b]SCOP type of targets.
[c]Average TM-score between the first I-TASSER model of design sequence and the structural analogs used for profiling.
[d]TM-score computed between the first I-TASSER model of design sequence and the target scaffold.
[e]RMSD computed between the first I-TASSER model of design sequence and the target scaffold.
