# Supplementary material for: An Evolution-Based Approach to De Novo Protein Design and Case Study on Mycobacterium tuberculosis
Source: PLoS Comput Biol. 2013 Oct 24;9(10):e1003298. doi: 10.1371/journal.pcbi.1003298 (PMC3812052; doi:10.1371/journal.pcbi.1003298)
Supplement: Table S3 — Summary of EvBM design on the 87 test proteins. (PDF) [file pcbi.1003298.s008.pdf]

Table S3. Summary of EvBM design on the 87 test proteins.

PDBID	TM-score[a]	RMSD (Å)[b]	Normalized relative error				Sequence identity (%)	
			SS	Φ	Ψ	SA	All	Core
1A2P_A	0.99	0.35	0.12	0.31	0.03	-0.03	48	44
1ABA_A	0.69	3.41	3.20	0.64	0.23	0.30	18	25
1BKR_A	0.99	0.28	0.33	0.09	-0.10	-0.05	42	55
1DBW_A	0.85	2.02	-0.08	0.14	0.00	0.00	29	44
1EAQ_A	0.92	1.42	-0.26	-0.03	-0.19	-0.11	30	37
1EW4_A	0.94	0.92	0.75	0.61	0.34	-0.07	16	31
1F46_A	0.97	0.66	0.27	0.25	-0.23	-0.01	27	29
1GBS_A	0.99	0.16	1.35	0.19	0.07	0.13	54	65
1GUT_A	0.91	0.77	-0.33	0.09	-0.12	0.03	24	11
1HZT_A	0.95	1.08	0.00	0.42	0.25	0.04	21	23
1I2T_A	0.93	0.76	0.29	0.12	0.69	0.20	25	57
1IDP_A	0.75	3.69	0.16	0.17	0.08	0.14	26	21
1IUJ_A	0.72	3.13	0.57	0.06	0.60	-0.19	27	43
1JB3_A	0.48	6.07	0.12	0.32	-0.10	0.28	26	33
1JF8_A	0.78	3.04	0.09	0.31	0.93	0.26	28	34
1KMT_A	0.95	1.04	0.14	0.10	0.01	-0.02	28	30
1KNG_A	0.96	0.95	0.50	0.17	0.11	0.03	26	34
1KQ1_A	0.81	1.65	0.29	0.79	0.44	0.08	23	54
1M9Z_A	0.92	1.26	0.19	0.22	0.68	0.01	45	51
1MF7_A	0.96	1.16	0.14	0.29	0.15	0.07	37	57
1MG4_A	0.45	8.43	1.67	0.45	0.52	0.14	13	20
1NXM_A	0.94	1.48	0.09	0.33	0.36	0.11	26	24
1NZ0_A	0.58	6.80	0.74	0.24	0.37	0.23	14	25
1O7I_A	0.99	0.39	-0.15	0.32	-0.17	0.02	22	33
1OAI_A	0.65	5.06	0.80	1.00	1.25	0.14	21	8
1OH0_A	0.96	0.95	0.05	0.27	0.00	0.04	31	35
1OK0_A	0.62	4.06	-0.14	0.05	-0.11	0.03	14	25
1QHQ_A	0.51	9.60	0.31	0.32	0.34	-0.07	30	40
1R26_A	0.90	1.33	0.20	0.21	-0.07	0.07	30	31
1R6J_A	0.91	1.48	-0.25	0.32	-0.06	0.07	32	50
1SHU_X	0.96	1.21	0.13	0.40	-0.05	-0.04	25	42
1T3Y_A	0.73	3.57	0.08	0.23	0.31	0.15	19	24
1TQG_A	0.94	0.96	1.25	0.04	0.16	-0.02	24	21
1TUK_A	0.25	11.13	0.44	0.52	2.10	0.02	10	16
1UCS_A	0.85	1.86	-0.16	-0.01	-0.12	-0.09	50	72
1URR_A	0.94	0.96	0.13	0.11	0.73	0.00	31	38
1UTG_A	0.83	2.08	3.33	-0.19	0.41	-0.29	10	10
1V5I_B	0.63	3.25	-0.29	0.34	0.03	-0.15	10	0
1VH5_A	0.98	0.48	0.13	0.41	0.22	0.06	31	28
1VKK_A	0.93	1.45	0.13	0.24	1.07	0.02	26	28
1VQS_A	0.68	3.31	-0.11	0.41	0.33	0.03	20	7
1VZI_A	0.34	11.67	0.52	0.43	-0.01	0.23	6	8
1WLU_A	0.98	0.49	0.00	0.31	0.08	0.03	32	41

1X6Z_A	0.67	4.23	0.53	0.52	0.42	-0.03	31	22
1XTE_A	0.89	2.06	0.27	0.25	0.42	-0.09	26	44
1ZHV_A	0.65	5.86	0.56	0.54	1.05	0.26	14	18
1ZKE_A	0.26	14.51	0.06	-0.03	0.01	0.14	12	18
1ZZK_A	0.83	3.11	0.29	0.48	0.16	0.03	41	64
2ANX_A	0.99	0.30	0.09	0.08	0.30	0.00	28	34
2BWF_A	0.92	1.21	-0.35	0.34	0.27	-0.02	40	65
2C9Q_A	0.77	2.51	0.39	0.28	0.16	-0.29	19	29
2CAR_A	0.96	1.06	-0.03	0.12	0.13	0.06	31	40
2CMP_A	0.61	2.27	4.00	0.34	0.36	0.00	17	23
2CVI_A	0.92	0.95	-0.17	0.32	0.58	0.03	29	36
2D3D_A	0.90	1.29	0.16	0.39	1.24	0.04	24	38
2ERB_A	0.99	0.35	0.50	0.18	0.27	0.01	32	39
2F01_A	0.98	0.75	-0.22	-0.08	-0.05	0.12	43	52
2FTR_A	0.73	2.73	0.44	0.62	0.42	0.05	27	24
2GMY_A	0.97	1.44	0.42	0.32	0.35	0.20	23	26
2GPI_A	0.46	8.26	0.93	0.42	0.42	-0.13	12	17
2J2J_A	0.78	3.54	-0.19	0.23	0.20	-0.14	21	31
2J5Y_A	0.93	0.86	3.00	-0.31	0.32	-0.04	33	36
2J8B_A	0.91	1.23	3.33	0.14	0.21	0.19	41	52
2O1Q_A	0.64	9.04	0.71	0.49	0.32	0.03	19	24
2O9S_A	0.81	3.53	0.25	0.25	-0.07	0.03	34	53
2P5K_A	0.93	0.73	0.33	0.17	1.12	0.28	30	38
2PR7_A	0.70	5.72	0.22	0.37	0.36	0.03	19	25
2PTH_A	0.96	1.37	0.29	0.29	-0.10	0.04	36	51
2PV2_A	0.95	0.84	0.36	0.10	0.19	0.23	33	53
2QCP_X	0.89	1.16	1.67	1.15	0.89	0.07	14	21
2V0U_A	0.93	2.74	0.05	0.10	0.24	0.07	59	68
2V1Q_A	0.88	1.11	0.20	0.25	0.33	0.05	29	29
2VMH_A	0.90	2.13	0.14	0.18	0.11	0.02	24	22
2VPB_A	0.83	1.27	0.07	-0.18	0.44	0.13	32	55
2VZC_A	0.91	1.48	0.24	0.43	0.79	0.15	20	34
2WLV_A	0.91	1.62	-0.11	0.10	0.25	-0.02	42	49
2ZXY_A	0.81	1.98	0.09	0.45	0.06	-0.04	33	41
3CTG_A	0.97	0.74	-0.12	0.15	-0.09	-0.02	43	51
3E9T_A	0.99	0.29	0.33	0.08	0.10	0.03	41	50
3EBT_A	0.89	1.63	0.00	0.18	0.15	0.04	29	28
3EF8_A	0.80	7.14	0.28	0.31	0.29	-0.06	28	33
3FEA_A	0.97	0.54	-0.12	0.45	0.24	0.04	35	58
3FIL_A	0.92	0.75	-0.11	-0.01	-0.21	0.01	52	75
3G21_A	0.29	15.25	2.71	0.15	1.27	-0.32	12	16
3G36_A	0.41	5.03	3.67	0.18	0.84	0.02	13	0
3IV4_A	0.81	2.11	-0.36	0.04	0.04	0.00	20	24
3VUB_A	0.71	3.10	0.38	0.20	0.11	0.11	20	31
Average	0.82	2.82	0.48	0.26	0.30	0.04	27	35

[a]TM-score between the first I-TASSER model of design sequence and the target scaffold.
[b]RMSD between the first I-TASSER model and the target scaffold.
