# Supplementary material for: An Evolution-Based Approach to De Novo Protein Design and Case Study on Mycobacterium tuberculosis
Source: PLoS Comput Biol. 2013 Oct 24;9(10):e1003298. doi: 10.1371/journal.pcbi.1003298 (PMC3812052; doi:10.1371/journal.pcbi.1003298)
Supplement: Table S4 — Summary of PBM design on the 87 test proteins. (PDF) [file pcbi.1003298.s009.pdf]

Table S4. Summary of PBM design on the 87 test proteins.

PDBID	TM-score[a]	RMSD (Å)[b]	Normalized relative error				Sequence Identity (%)	
			SS	Φ	Ψ	SA	All	Core
1A2P_A	0.91	1.45	2.88	0.23	1.11	0.73	21	38
1ABA_A	0.94	0.91	0.80	-0.07	0.01	0.05	33	58
1BKR_A	0.98	0.45	2.22	0.09	0.21	0.48	24	40
1DBW_A	0.90	1.49	3.17	0.44	0.92	0.34	20	27
1EAQ_A	0.89	1.53	0.46	-0.08	0.24	-0.04	23	37
1EW4_A	0.26	11.88	2.58	0.45	1.04	0.30	12	7
1F46_A	0.98	0.52	0.18	0.07	0.07	0.15	23	33
1GBS_A	0.87	2.42	3.00	0.56	0.30	0.65	19	27
1GUT_A	0.76	1.49	3.17	-0.01	0.67	0.08	20	44
1HZT_A	0.97	0.79	1.17	1.03	2.58	0.54	23	33
1I2T_A	0.74	1.79	0.29	-0.14	0.23	0.33	15	21
1IDP_A	0.20	16.66	2.13	0.54	1.70	0.61	17	30
1IUJ_A	0.63	4.21	2.86	0.26	2.92	0.81	19	21
1JB3_A	0.98	0.57	0.92	0.06	0.11	0.07	25	39
1JF8_A	0.99	0.36	2.18	0.46	1.40	0.42	25	44
1KMT_A	0.29	10.24	3.03	0.75	2.11	0.45	21	32
1KNG_A	0.99	0.27	1.88	0.69	1.55	0.43	24	28
1KQ1_A	0.21	14.84	5.57	1.27	2.47	0.34	17	31
1M9Z_A	0.18	15.92	3.38	0.64	2.02	0.46	8	8
1MF7_A	0.92	1.68	1.89	0.74	1.16	0.70	14	20
1MG4_A	0.84	2.28	3.50	0.34	0.44	0.40	24	37
1NXM_A	0.89	2.56	1.79	0.47	1.20	0.62	18	19
1NZ0_A	0.66	4.18	1.16	0.05	0.16	0.74	23	38
1O7I_A	0.98	0.48	2.77	0.88	1.16	0.35	26	44
1OAI_A	0.85	1.37	1.00	1.09	2.00	0.37	18	23
1OH0_A	0.95	1.12	2.47	1.52	3.17	0.65	24	24
1OK0_A	0.72	2.27	0.32	-0.12	0.22	0.21	27	55
1QHQ_A	0.98	0.47	0.76	0.18	0.86	0.27	21	33
1R26_A	0.75	2.78	9.80	1.36	3.33	0.47	16	23
1R6J_A	0.88	1.51	3.75	0.30	1.27	0.44	32	65
1SHU_X	0.96	1.17	0.25	0.09	-0.14	0.04	21	24
1T3Y_A	0.19	15.86	1.65	0.58	0.73	0.54	17	33
1TQG_A	0.41	6.43	1.50	0.53	0.76	0.83	13	18
1TUK_A	0.77	1.75	-0.11	0.01	0.17	-0.04	25	42
1UCS_A	0.96	0.81	0.05	0.13	0.75	0.45	30	61
1URR_A	0.96	0.87	4.25	0.67	2.70	0.49	23	44
1UTG_A	0.92	0.86	1.00	-0.21	0.48	0.22	17	30

1V5I_B	0.64	3.66	1.36	0.44	0.95	0.36	26	50
1VH5_A	0.96	0.87	7.00	0.83	2.00	0.26	20	30
1VKK_A	0.21	17.15	3.13	0.63	2.81	0.57	16	28
1VQS_A	0.70	5.75	1.05	0.58	1.66	0.48	23	25
1VZI_A	0.97	0.69	1.74	0.40	0.94	0.40	22	42
1WLU_A	0.97	0.75	9.00	2.12	4.41	0.40	25	38
1X6Z_A	0.91	1.47	1.65	0.27	0.37	0.27	24	27
1XTE_A	0.85	2.90	3.09	0.30	1.50	0.37	17	19
1ZHV_A	0.98	0.64	1.40	0.46	1.56	0.38	29	49
1ZKE_A	0.90	1.95	-0.39	-0.27	-0.61	0.72	22	29
1ZZK_A	0.84	2.85	1.29	0.20	0.02	0.59	26	45
2ANX_A	0.88	3.04	0.83	0.43	0.56	0.74	21	40
2BWF_A	0.87	1.35	0.65	0.71	2.77	0.26	17	35
2C9Q_A	0.78	2.56	2.26	0.34	1.55	0.62	25	39
2CAR_A	0.99	0.32	-0.03	-0.08	-0.03	-0.61	25	39
2CMP_A	0.82	1.27	5.50	0.42	1.42	0.54	25	69
2CVI_A	0.34	9.84	3.08	1.37	3.11	0.58	23	50
2D3D_A	0.90	1.12	0.26	0.26	0.46	0.87	25	54
2ERB_A	0.87	2.84	3.33	0.20	0.76	0.67	19	22
2F01_A	0.44	6.69	1.89	0.51	1.18	0.45	19	21
2FTR_A	0.32	9.23	3.00	0.42	0.83	0.39	23	28
2GMY_A	0.95	1.17	2.42	0.72	1.39	0.58	15	26
2GPI_A	0.25	13.64	2.50	0.35	0.95	0.78	16	35
2J2J_A	0.17	17.86	1.65	0.09	0.84	0.14	20	34
2J5Y_A	0.37	11.98	11.00	0.04	-0.07	0.43	13	36
2J8B_A	0.20	9.20	11.33	0.49	0.88	0.41	9	12
2O1Q_A	0.97	0.92	0.17	0.09	0.71	-0.14	28	33
2O9S_A	0.80	3.24	3.62	0.31	1.33	0.61	34	63
2P5K_A	0.25	11.87	5.33	1.11	3.81	0.38	19	38
2PR7_A	0.98	0.61	-0.44	-0.32	-0.11	-0.05	30	33
2PTH_A	0.94	1.37	0.52	0.18	0.12	0.11	22	29
2PV2_A	0.97	0.66	2.09	1.04	1.22	0.67	24	44
2QCP_X	0.95	0.74	4.33	0.74	1.09	0.34	26	63
2V0U_A	0.93	4.26	-0.50	0.21	-0.04	0.10	17	26
2V1Q_A	0.86	1.13	5.60	0.88	1.81	0.51	27	64
2VMH_A	0.77	3.10	2.95	0.27	1.29	0.64	23	31
2VPB_A	0.21	9.99	0.43	-0.09	0.64	0.41	11	18
2VZC_A	0.99	0.27	0.06	-0.01	0.07	-0.05	28	43
2WLV_A	0.97	0.83	0.89	0.05	0.30	0.42	20	27
2ZXY_A	0.20	12.48	2.27	0.04	-0.26	0.64	20	27
3CTG_A	0.97	0.69	0.81	0.73	0.82	0.36	25	35

3E9T_A	0.98	0.60	6.67	0.43	1.67	0.57	22	41
3EBT_A	0.67	4.54	3.44	1.84	2.66	0.70	27	47
3EF8_A	0.22	18.02	2.76	0.93	1.97	0.48	17	29
3FEA_A	0.97	0.51	0.94	0.45	0.66	0.62	34	58
3FIL_A	0.89	0.93	1.89	-0.03	0.30	0.49	25	58
3G21_A	0.36	8.68	2.00	0.10	0.42	0.77	16	5
3G36_A	0.52	4.50	1.00	-0.14	0.52	0.03	15	0
3IV4_A	0.79	2.46	1.00	0.39	0.75	0.17	14	26
3VUB_A	0.91	1.17	1.03	0.13	0.47	0.24	19	34
Average	0.74	4.14	2.40	0.43	1.11	0.41	21	35

[a]TM-score between the first I-TASSER model of design sequence and the target scaffold.
[b]RMSD between the first I-TASSER model and the target scaffold.
