# Supplementary material for: An Evolution-Based Approach to De Novo Protein Design and Case Study on Mycobacterium tuberculosis
Source: PLoS Comput Biol. 2013 Oct 24;9(10):e1003298. doi: 10.1371/journal.pcbi.1003298 (PMC3812052; doi:10.1371/journal.pcbi.1003298)
Supplement: Table S5 — Summary of EBM based protein design on the M. tuberculosis genome. The data has been sorted by the normalized relative error (NRE) on secondary structure. (PDF) [file pcbi.1003298.s010.pdf]

**Table S5.** Summary of EBM based protein design on the *M. tuberculosis* genome. The data has been sorted by the normalized relative error (NRE) on secondary structure.

| PDBID | L[a] | Scp[b] | Np[c] | SI%[d] | R(Å)[e] | Normalized relative error | | | | Sequence identity (%) | |
|---|---|---|---|---|---|---|---|---|---|---|---|
| | | | | | | SS | Φ | Ψ | SA | All | Core |
| 3FAV_A | 74 | | 167 | 9 | 2.14 | -0.47 | -0.54 | -0.64 | -0.47 | 9 | 17 |
| 3FAV_B | 72 | | 34 | 10 | 2.34 | -0.40 | -0.32 | 0.08 | 0.03 | 17 | 18 |
| 2G38_A | 77 | a | 60 | 10 | 2.46 | -0.36 | -0.09 | -0.25 | -0.71 | 18 | 58 |
| 3H6P_C | 56 | | 20 | 13 | 3.23 | -0.33 | 0.12 | 0.13 | -0.01 | 14 | 20 |
| 1IM5_A | 179 | a/b | 22 | 25 | 0.28 | -0.29 | 0.03 | 0.01 | -0.02 | 39 | 59 |
| 2ZHX_B | 82 | a+b | 338 | 7 | 0.89 | -0.28 | 0.11 | 0.21 | -0.05 | 17 | 20 |
| 1P3H_A | 99 | b | 53 | 17 | 1.06 | -0.27 | 0.07 | -0.07 | 0.06 | 26 | 47 |
| 3KNH_H | 138 | | 10 | 41 | 1.89 | -0.26 | 0.06 | -0.12 | 0.00 | 46 | 44 |
| 3KNI_S | 98 | | 27 | 15 | 3.57 | -0.25 | -0.07 | -0.08 | 0.23 | 17 | 27 |
| 2PMU_A | 98 | | 11 | 30 | 1.44 | -0.25 | 0.05 | 0.46 | 0.03 | 36 | 50 |
| 1UVH_A | 157 | a | 88 | 13 | 2.42 | -0.23 | -0.11 | -0.03 | -0.05 | 22 | 28 |
| 3MPZ_A | 125 | | 11 | 38 | 0.42 | -0.20 | 0.23 | 0.39 | 0.08 | 50 | 55 |
| 3NFW_A | 179 | | 20 | 20 | 0.28 | -0.19 | -0.05 | 0.02 | -0.02 | 30 | 51 |
| 3M6C_A | 194 | | 30 | 16 | 0.87 | -0.19 | 0.01 | 0.10 | 0.07 | 31 | 44 |
| 2W72_B | 146 | | 71 | 25 | 0.89 | -0.19 | -0.16 | -0.09 | 0.00 | 30 | 38 |
| 3IOS_A | 134 | | 93 | 18 | 2.02 | -0.18 | 0.07 | 0.10 | 0.03 | 28 | 39 |
| 2CGQ_A | 74 | | 31 | 23 | 1.24 | -0.18 | -0.23 | -0.01 | -0.06 | 35 | 62 |
| 1TFU_A | 157 | a/b | 28 | 22 | 0.91 | -0.16 | -0.03 | -0.06 | 0.01 | 36 | 47 |
| 2A6P_A | 193 | | 22 | 23 | 2.65 | -0.15 | 0.01 | -0.08 | 0.17 | 30 | 41 |
| 2H7V_A | 178 | a/b | 95 | 24 | 1.43 | -0.15 | 0.05 | 0.36 | -0.01 | 33 | 38 |
| 2NM3_A | 121 | a+b | 13 | 20 | 0.33 | -0.14 | 0.02 | -0.01 | 0.03 | 31 | 50 |
| 3RIH_A | 260 | | 224 | 25 | 0.85 | -0.14 | 0.06 | -0.11 | 0.02 | 33 | 42 |
| 3HX9_A | 101 | | 16 | 16 | 1.15 | -0.14 | 0.21 | 0.41 | -0.09 | 27 | 31 |
| 2Z2I_A | 179 | a+b | 60 | 12 | 1.75 | -0.13 | 0.21 | 0.20 | 0.03 | 16 | 19 |
| 3NY4_A | 265 | | 48 | 24 | 1.45 | -0.13 | 0.13 | 0.10 | 0.01 | 26 | 38 |
| 3P0T_A | 136 | | 10 | 21 | 1.00 | -0.12 | 0.06 | 0.06 | -0.03 | 30 | 55 |
| 3KNI_D | 271 | | 10 | 36 | 1.89 | -0.12 | 0.09 | 0.14 | -0.05 | 37 | 47 |
| 3NBK_A | 158 | | 30 | 25 | 0.91 | -0.12 | -0.01 | 0.05 | -0.02 | 39 | 53 |
| 3P2J_A | 191 | | 44 | 15 | 1.22 | -0.12 | 0.01 | 0.27 | -0.01 | 27 | 34 |
| 2Z90_A | 161 | | 72 | 13 | 6.67 | -0.12 | -0.12 | 0.08 | -0.07 | 17 | 21 |
| 2VKL_A | 77 | | 22 | 12 | 3.26 | -0.11 | 0.22 | -0.12 | 0.18 | 12 | 33 |
| 1U2P_A | 156 | | 12 | 27 | 1.09 | -0.10 | 0.13 | -0.12 | 0.01 | 37 | 52 |
| 3MYB_A | 259 | | 66 | 23 | 2.03 | -0.09 | 0.04 | 0.05 | 0.04 | 28 | 44 |
| 3KNH_E | 150 | | 10 | 27 | 1.28 | -0.09 | 0.13 | 0.12 | -0.01 | 35 | 48 |
| 2YW6_A | 148 | a | 98 | 14 | 1.52 | -0.09 | -0.13 | -0.21 | -0.11 | 20 | 20 |
| 3A2G_A | 154 | | 72 | 22 | 0.95 | -0.08 | 0.26 | 0.08 | -0.02 | 34 | 37 |
| 2QXX_A | 189 | | 12 | 19 | 1.02 | -0.08 | 0.30 | 0.13 | 0.04 | 32 | 45 |
| 2XWL_A | 221 | | 50 | 18 | 0.42 | -0.08 | 0.24 | -0.07 | -0.01 | 23 | 32 |
| 3MXU_A | 134 | | 11 | 28 | 4.36 | -0.08 | -0.07 | -0.09 | 0.17 | 35 | 47 |
| 3QXZ_A | 259 | | 66 | 23 | 1.89 | -0.07 | 0.06 | 0.13 | 0.09 | 32 | 47 |
| 3KNH_G | 155 | | 10 | 40 | 3.80 | -0.07 | 0.27 | 0.14 | 0.20 | 37 | 48 |

| | | | | | | | | | | | |
|---|---|---|---|---|---|---|---|---|---|---|---|
| 3KNH_I | 127 | | 14 | 28 | 1.07 | -0.07 | -0.05 | -0.01 | 0.00 | 38 | 38 |
| 3R9T_A | 260 | | 70 | 26 | 18.09 | -0.06 | 0.04 | -0.03 | 0.00 | 31 | 50 |
| 1J7S_B | 146 | a | 71 | 26 | 0.94 | -0.06 | -0.05 | -0.10 | 0.00 | 36 | 46 |
| 2VVR_A | 147 | | 11 | 41 | 0.86 | -0.06 | -0.12 | 0.03 | 0.01 | 44 | 57 |
| 2C45_A | 113 | | 66 | 13 | 0.52 | -0.06 | -0.09 | -0.19 | -0.01 | 24 | 34 |
| 3MOY_A | 258 | | 67 | 27 | 1.15 | -0.06 | 0.04 | 0.10 | 0.03 | 34 | 51 |
| 2A15_A | 133 | a+b | 47 | 15 | 0.91 | -0.05 | 0.09 | 0.48 | -0.07 | 22 | 15 |
| 1ZNW_A | 182 | a/b | 12 | 31 | 0.55 | -0.05 | 0.08 | 0.04 | -0.04 | 37 | 55 |
| 3KNI_6 | 50 | | 22 | 19 | 5.20 | -0.05 | -0.04 | -0.13 | -0.06 | 12 | 20 |
| 3C4I_A | 99 | | 15 | 26 | 0.51 | -0.05 | -0.13 | -0.09 | -0.01 | 37 | 60 |
| 3HGB_A | 134 | | 10 | 43 | 0.35 | -0.05 | -0.02 | -0.07 | -0.01 | 50 | 65 |
| 3KNI_Y | 100 | | 10 | 27 | 4.79 | -0.04 | -0.10 | 0.08 | 0.04 | 34 | 56 |
| 3H81_A | 256 | | 69 | 28 | 1.04 | -0.04 | 0.17 | -0.04 | -0.01 | 36 | 55 |
| 3HH2_A | 109 | | 10 | 34 | 0.60 | -0.03 | 0.12 | -0.17 | 0.07 | 39 | 78 |
| 1YK9_A | 184 | | 13 | 28 | 7.79 | -0.03 | 0.08 | 0.29 | 0.35 | 24 | 39 |
| 3KNI_E | 204 | | 11 | 30 | 10.20 | -0.03 | 0.21 | 0.13 | 0.63 | 15 | 20 |
| 2QZ8_A | 146 | | 10 | 32 | 1.34 | -0.03 | 0.02 | -0.11 | 0.02 | 36 | 53 |
| 2NTV_A | 268 | | 188 | 18 | 2.85 | -0.03 | 0.19 | 0.15 | 0.00 | 24 | 27 |
| 1P9L_A | 245 | a/b | 107 | 12 | 0.96 | -0.03 | 0.43 | 0.31 | 0.04 | 19 | 26 |
| 3FGN_A | 230 | | 13 | 16 | 0.50 | -0.03 | 0.32 | -0.02 | 0.00 | 20 | 31 |
| 3GWK_C | 98 | | 17 | 17 | 1.29 | 0.00 | -0.23 | 0.10 | -0.17 | 30 | 54 |
| 3KNH_J | 98 | | 10 | 35 | 1.24 | 0.00 | -0.12 | -0.14 | 0.04 | 40 | 55 |
| 2X89_A | 128 | | 105 | 37 | 2.35 | 0.00 | -0.04 | -0.17 | 0.03 | 52 | 58 |
| 3KNI_R | 117 | | 20 | 17 | 1.52 | 0.00 | -0.08 | -0.11 | -0.01 | 29 | 41 |
| 3EUL_A | 123 | | 178 | 18 | 0.74 | 0.00 | 0.00 | -0.11 | 0.00 | 33 | 45 |
| 3KNI_W | 113 | | 10 | 37 | 0.89 | 0.00 | -0.09 | 0.13 | -0.01 | 43 | 56 |
| 3C57_A | 51 | | 19 | 28 | 6.53 | 0.00 | -0.24 | -0.31 | 0.06 | 29 | 63 |
| 2C2X_A | 280 | | 48 | 16 | 1.16 | 0.00 | 0.02 | 0.00 | 0.04 | 26 | 35 |
| 2W29_A | 147 | | 10 | 32 | 0.92 | 0.00 | -0.04 | 0.04 | 0.03 | 37 | 47 |
| 3HUG_A | 80 | | 22 | 13 | 3.00 | 0.00 | 0.40 | 0.12 | 0.11 | 26 | 75 |
| 1L4U_A | 165 | a/b | 61 | 17 | 3.02 | 0.00 | 0.01 | -0.04 | -0.02 | 21 | 29 |
| 3LHD_A | 253 | | 21 | 19 | 0.80 | 0.00 | 0.33 | 0.40 | -0.01 | 25 | 30 |
| 3R20_A | 217 | | 10 | 27 | 0.17 | 0.00 | 0.29 | -0.10 | 0.04 | 36 | 52 |
| 1W66_A | 218 | a+b | 14 | 14 | 0.25 | 0.00 | 0.09 | 0.03 | 0.05 | 23 | 30 |
| 2O8X_A | 61 | | 16 | 22 | 1.66 | 0.00 | 0.19 | -0.01 | -0.19 | 30 | 45 |
| 3KNI_2 | 71 | | 31 | 17 | 0.86 | 0.00 | 0.04 | -0.28 | 0.02 | 23 | 70 |
| 1WQG_A | 184 | a+b | 10 | 24 | 1.45 | 0.00 | 0.20 | -0.01 | 0.00 | 40 | 48 |
| 1XZ0_B | 98 | b | 119 | 20 | 1.96 | 0.00 | 0.04 | -0.06 | 0.31 | 29 | 50 |
| 2W6X_A | 154 | | 72 | 22 | 0.93 | 0.00 | 0.06 | 0.14 | 0.03 | 31 | 38 |
| 1W19_A | 147 | | 10 | 36 | 0.59 | 0.00 | 0.06 | 0.12 | 0.00 | 40 | 54 |
| 3KNI_X | 92 | | 10 | 35 | 1.60 | 0.00 | 0.06 | -0.13 | -0.03 | 36 | 48 |
| 3IJF_X | 123 | | 10 | 35 | 0.82 | 0.00 | 0.21 | 0.38 | -0.05 | 40 | 50 |
| 3D55_A | 68 | | 19 | 12 | 1.40 | 0.00 | 0.17 | 0.34 | 0.26 | 16 | 17 |
| 2I1U_A | 108 | | 137 | 24 | 2.52 | 0.00 | 0.27 | 0.52 | 0.00 | 38 | 65 |
| 1NXJ_A | 156 | a/b | 30 | 16 | 0.22 | 0.00 | 0.10 | 0.30 | 0.04 | 35 | 49 |
| 1DF7_A | 159 | a/b | 32 | 27 | 0.27 | 0.00 | 0.16 | 0.05 | 0.04 | 34 | 46 |
| 2FVH_A | 99 | | 86 | 11 | 0.93 | 0.00 | 0.22 | 0.23 | -0.01 | 23 | 39 |
| 3ICO_A | 244 | | 15 | 29 | 0.30 | 0.03 | 0.01 | 0.08 | 0.04 | 31 | 46 |
| 2W3B_A | 185 | | 29 | 27 | 0.82 | 0.03 | 0.11 | 0.20 | 0.01 | 38 | 51 |

| | | | | | | | | | | | | |
|---|---|---|---|---|---|---|---|---|---|---|---|---|
| 3NDO_A | 225 | | 252 | 12 | 2.58 | 0.03 | 0.07 | 0.02 | 0.01 | 23 | 29 |
| 3QKA_A | 233 | | 71 | 25 | 2.24 | 0.03 | 0.19 | -0.07 | 0.06 | 33 | 47 |
| 2W25_A | 147 | | 10 | 25 | 1.24 | 0.03 | -0.01 | -0.01 | 0.04 | 29 | 41 |
| 1U5H_A | 223 | a/b | 68 | 13 | 0.45 | 0.03 | 0.11 | -0.03 | 0.01 | 23 | 29 |
| 2XB9_A | 158 | | 202 | 10 | 0.98 | 0.04 | -0.02 | 0.32 | 0.09 | 20 | 32 |
| 3KNI_U | 117 | | 14 | 24 | 1.27 | 0.04 | -0.05 | -0.09 | -0.01 | 38 | 56 |
| 1Y25_A | 165 | a/b | 42 | 22 | 1.03 | 0.04 | 0.23 | 0.00 | 0.09 | 29 | 38 |
| 1NBU_A | 118 | a+b | 18 | 23 | 0.76 | 0.05 | -0.12 | -0.07 | -0.05 | 33 | 59 |
| 1NBU_C | 118 | a+b | 16 | 19 | 0.72 | 0.05 | -0.02 | -0.04 | 0.00 | 32 | 49 |
| 3KNH_N | 60 | | 10 | 21 | 2.36 | 0.05 | -0.04 | 0.01 | 0.10 | 33 | 57 |
| 3GWM_A | 129 | | 13 | 27 | 3.41 | 0.05 | 0.35 | 0.18 | 0.07 | 34 | 50 |
| 3KNH_B | 234 | | 27 | 14 | 1.84 | 0.05 | 0.00 | -0.05 | -0.02 | 19 | 31 |
| 2JFR_A | 234 | | 14 | 22 | 3.52 | 0.05 | 0.29 | 0.37 | 0.09 | 22 | 34 |
| 1J7Y_B | 146 | a | 71 | 25 | 0.85 | 0.06 | -0.06 | -0.17 | 0.00 | 30 | 38 |
| 3I86_A | 136 | | 10 | 38 | 1.02 | 0.06 | -0.05 | 0.02 | 0.06 | 49 | 62 |
| 3KNI_V | 101 | | 12 | 12 | 4.53 | 0.07 | -0.03 | 0.14 | 0.54 | 22 | 43 |
| 3KNH_F | 101 | | 95 | 10 | 4.27 | 0.07 | 0.19 | 0.71 | -0.03 | 21 | 41 |
| 2Y88_A | 244 | | 46 | 15 | 4.99 | 0.07 | 0.25 | 0.05 | 0.04 | 23 | 28 |
| 2CDN_A | 186 | a/b | 26 | 28 | 1.22 | 0.07 | 0.13 | 0.29 | 0.04 | 36 | 53 |
| 3I7T_A | 118 | | 26 | 26 | 1.58 | 0.07 | -0.06 | -0.03 | 0.08 | 36 | 60 |
| 3NE3_B | 129 | | 12 | 29 | 2.30 | 0.07 | 0.26 | -0.09 | 0.04 | 33 | 46 |
| 1NAZ_A | 154 | a | 71 | 22 | 1.12 | 0.08 | 0.39 | 0.23 | 0.07 | 24 | 29 |
| 3PK0_A | 260 | | 223 | 25 | 0.63 | 0.08 | 0.02 | 0.09 | 0.02 | 26 | 37 |
| 3QHA_A | 277 | | 52 | 16 | 14.76 | 0.08 | 0.19 | -0.05 | 0.05 | 24 | 38 |
| 2A11_A | 154 | | 10 | 24 | 0.93 | 0.08 | 0.11 | -0.24 | -0.03 | 37 | 51 |
| 3ASE_A | 154 | | 71 | 22 | 0.89 | 0.08 | 0.17 | 0.08 | 0.02 | 34 | 43 |
| 2IVM_A | 147 | | 10 | 33 | 1.01 | 0.09 | -0.02 | -0.03 | 0.04 | 39 | 56 |
| 3ONR_A | 71 | | 14 | 14 | 2.44 | 0.09 | 0.24 | 0.13 | 0.05 | 27 | 78 |
| 3KNI_F | 207 | | 10 | 30 | 5.10 | 0.09 | 0.03 | 0.18 | 0.02 | 28 | 31 |
| 3HZA_A | 162 | | 11 | 32 | 0.27 | 0.10 | 0.07 | 0.22 | -0.01 | 41 | 64 |
| 3SBX_A | 177 | | 11 | 27 | 0.36 | 0.10 | -0.05 | 0.45 | 0.00 | 31 | 39 |
| 2CHC_A | 169 | a+b | 42 | 12 | 4.03 | 0.10 | 0.37 | 0.19 | -0.04 | 24 | 33 |
| 3PL1_A | 185 | | 20 | 25 | 5.31 | 0.10 | 0.19 | 0.20 | 0.27 | 22 | 32 |
| 3QJ7_A | 260 | | 17 | 47 | 0.82 | 0.10 | 0.11 | 0.04 | 0.08 | 41 | 49 |
| 3ODG_A | 273 | | 25 | 23 | 1.57 | 0.11 | 0.22 | 0.15 | 0.08 | 34 | 40 |
| 2V2X_A | 275 | b | 19 | 36 | 7.19 | 0.11 | 0.34 | 0.18 | 0.37 | 25 | 29 |
| 3HNT_L | 214 | | 17 | 41 | 3.60 | 0.11 | 0.13 | 0.05 | 0.16 | 38 | 55 |
| 3L60_A | 229 | | 24 | 15 | 1.06 | 0.11 | 0.15 | 0.24 | 0.03 | 21 | 30 |
| 3KNI_O | 122 | | 10 | 56 | 3.65 | 0.11 | 0.15 | 0.01 | 0.28 | 50 | 55 |
| 1J7W_A | 141 | a | 74 | 27 | 0.83 | 0.13 | 0.08 | 0.05 | -0.07 | 38 | 53 |
| 1Y0H_A | 101 | a+b | 36 | 15 | 1.19 | 0.13 | 0.04 | 0.28 | 0.13 | 18 | 23 |
| 2WT9_A | 207 | | 17 | 23 | 0.32 | 0.13 | 0.19 | 0.05 | 0.03 | 29 | 44 |
| 3KNH_P | 83 | | 10 | 19 | 0.84 | 0.13 | 0.05 | 0.06 | 0.02 | 24 | 30 |
| 2R5U_A | 144 | | 20 | 13 | 0.40 | 0.13 | 0.27 | 0.13 | -0.01 | 28 | 48 |
| 3F8L_A | 162 | | 14 | 18 | 0.38 | 0.14 | 0.29 | 0.10 | 0.05 | 21 | 28 |
| 1D0I_A | 150 | a/b | 495 | 10 | 1.07 | 0.15 | 0.16 | 0.00 | 0.11 | 19 | 30 |
| 3HSS_A | 274 | | 79 | 16 | 0.69 | 0.15 | 0.09 | 0.08 | 0.00 | 21 | 31 |
| 2VE6_B | 99 | b | 120 | 19 | 1.63 | 0.15 | 0.03 | 0.16 | 0.02 | 33 | 55 |
| 2WUG_A | 283 | | 75 | 19 | 1.32 | 0.16 | 0.27 | 0.26 | 0.03 | 26 | 33 |

| | | | | | | | | | | | |
|---|---|---|---|---|---|---|---|---|---|---|---|
| 3R2N_A | 119 | | 11 | 32 | 0.48 | 0.17 | 0.26 | 0.39 | 0.03 | 45 | 52 |
| 3QK8_A | 259 | | 70 | 22 | 17.19 | 0.17 | 0.18 | 0.39 | 0.05 | 25 | 33 |
| 3I4O_A | 68 | | 21 | 20 | 1.67 | 0.17 | -0.09 | 0.11 | -0.17 | 32 | 58 |
| 1X89_A | 174 | b | 19 | 23 | 2.46 | 0.18 | 0.04 | 0.42 | 0.04 | 28 | 52 |
| 3KNI_Q | 141 | | 10 | 24 | 2.64 | 0.19 | 0.00 | 0.03 | -0.03 | 40 | 61 |
| 1L1E_A | 272 | a/b | 33 | 16 | 1.77 | 0.19 | 0.01 | 0.11 | 0.02 | 24 | 27 |
| 3H5J_A | 168 | | 12 | 25 | 1.26 | 0.19 | -0.12 | -0.16 | 0.01 | 36 | 47 |
| 1GTV_A | 208 | a/b | 17 | 20 | 2.38 | 0.20 | 0.44 | 0.10 | 0.03 | 27 | 41 |
| 1LU4_A | 134 | a/b | 86 | 16 | 1.93 | 0.20 | 0.28 | 0.31 | 0.03 | 23 | 40 |
| 3K3C_A | 152 | | 73 | 10 | 2.55 | 0.20 | 0.16 | 0.06 | 0.06 | 22 | 29 |
| 2VE6_A | 276 | a+b | 19 | 35 | 10.65 | 0.21 | 0.35 | 0.27 | 0.41 | 25 | 33 |
| 1TPY_A | 285 | a/b | 22 | 18 | 1.14 | 0.21 | 0.01 | 0.05 | 0.03 | 24 | 29 |
| 2BM5_A | 183 | b | 21 | 12 | 4.28 | 0.21 | 0.15 | 0.22 | 0.01 | 17 | 26 |
| 3TDT_A | 274 | b | 10 | 22 | 3.09 | 0.21 | 0.14 | -0.02 | 0.10 | 36 | 44 |
| 2GLN_A | 128 | | 85 | 14 | 1.03 | 0.22 | 0.09 | 0.18 | -0.04 | 32 | 50 |
| 2QRW_A | 126 | | 76 | 11 | 0.65 | 0.23 | 0.29 | -0.12 | 0.15 | 18 | 30 |
| 3KNI_N | 138 | | 11 | 39 | 1.12 | 0.24 | -0.02 | 0.09 | 0.01 | 41 | 60 |
| 3F8M_A | 230 | | 16 | 16 | 10.65 | 0.25 | 0.15 | 0.35 | 0.05 | 23 | 26 |
| 2V2X_B | 100 | b | 113 | 20 | 1.61 | 0.25 | -0.01 | -0.16 | 0.10 | 29 | 44 |
| 2B70_A | 162 | a+b | 13 | 88 | 4.53 | 0.25 | 0.39 | 0.60 | 0.34 | 46 | 41 |
| 3NE0_A | 208 | | 10 | 33 | 0.65 | 0.26 | 0.11 | 0.10 | 0.08 | 50 | 69 |
| 3PPI_A | 258 | | 221 | 22 | 1.04 | 0.26 | 0.11 | 0.16 | 0.03 | 29 | 40 |
| 3NB5_A | 257 | | 21 | 44 | 3.42 | 0.26 | 0.24 | 0.20 | 0.15 | 39 | 50 |
| 3DWG_C | 92 | | 13 | 19 | 1.29 | 0.27 | -0.19 | -0.23 | 0.01 | 37 | 50 |
| 2G4R_A | 153 | | 14 | 28 | 1.14 | 0.27 | -0.09 | 0.07 | -0.01 | 33 | 43 |
| 1PQW_A | 183 | a/b | 62 | 20 | 2.88 | 0.27 | 0.13 | -0.05 | 0.06 | 27 | 35 |
| 2O03_A | 129 | | 10 | 16 | 9.12 | 0.29 | 0.16 | -0.02 | 0.08 | 27 | 52 |
| 3RFQ_A | 154 | | 14 | 28 | 0.23 | 0.32 | 0.16 | 0.29 | 0.01 | 38 | 51 |
| 3QUA_A | 179 | | 11 | 28 | 2.64 | 0.33 | 0.23 | 0.49 | 0.12 | 31 | 39 |
| 3OCC_A | 237 | | 37 | 23 | 1.49 | 0.33 | 0.16 | 0.08 | -0.01 | 27 | 29 |
| 3PO8_A | 98 | | 30 | 23 | 1.12 | 0.33 | 0.10 | 0.09 | 0.03 | 29 | 39 |
| 3KNI_P | 146 | | 10 | 14 | 4.51 | 0.33 | 0.12 | 0.06 | 0.20 | 21 | 20 |
| 2XW7_A | 175 | | 30 | 15 | 4.62 | 0.34 | 0.51 | 0.29 | 0.15 | 20 | 29 |
| 1N3N_B | 99 | b | 118 | 19 | 2.70 | 0.37 | 0.04 | 0.43 | 0.20 | 25 | 37 |
| 3G1M_A | 194 | | 48 | 16 | 1.43 | 0.38 | 0.07 | 0.08 | -0.01 | 18 | 21 |
| 2XPG_A | 274 | | 19 | 22 | 12.09 | 0.38 | 0.19 | 0.38 | 0.37 | 29 | 34 |
| 2QVB_A | 296 | | 59 | 17 | 5.08 | 0.39 | 0.02 | -0.03 | 0.27 | 15 | 19 |
| 3OC6_A | 243 | | 15 | 29 | 4.42 | 0.40 | 0.06 | 0.41 | 0.27 | 21 | 32 |
| 3AJX_A | 207 | | 632 | 12 | 0.49 | 0.43 | 0.34 | 0.05 | 0.02 | 19 | 24 |
| 1QPO_A | 284 | a/b | 15 | 32 | 3.22 | 0.43 | 0.31 | 0.41 | 0.34 | 29 | 36 |
| 3D0S_A | 224 | | 10 | 31 | 4.74 | 0.45 | 0.24 | 0.54 | 0.06 | 27 | 38 |
| 1UZR_A | 282 | a | 20 | 21 | 2.84 | 0.46 | 0.01 | 0.32 | 0.37 | 18 | 26 |
| 3KNI_H | 163 | | 10 | 32 | 7.20 | 0.50 | 0.00 | 0.05 | 0.26 | 26 | 29 |
| 2VG0_A | 227 | | 44 | 9 | 1.74 | 0.52 | 0.12 | 0.29 | 0.12 | 21 | 25 |
| 2WZM_A | 274 | | 33 | 32 | 4.80 | 0.53 | 0.31 | 0.28 | 0.19 | 24 | 34 |
| 3R0O_A | 262 | | 68 | 25 | 18.90 | 0.54 | 0.34 | 0.38 | 0.18 | 24 | 32 |
| 2VVP_A | 157 | a/b | 10 | 27 | 3.62 | 0.55 | 0.53 | 0.57 | 0.01 | 32 | 42 |
| 1DT0_A | 197 | a+b | 34 | 45 | 4.75 | 0.55 | 0.29 | 0.32 | 0.47 | 31 | 39 |
| 2IB0_A | 142 | | 98 | 11 | 3.88 | 0.56 | 0.16 | 0.29 | 0.01 | 14 | 25 |

| | | | | | | | | | | | |
|---|---|---|---|---|---|---|---|---|---|---|---|
| 1W74_A | 171 | b | 30 | 37 | 3.63 | 0.56 | 0.26 | 0.13 | 0.15 | 33 | 46 |
| 2FR2_A | 161 | b | 18 | 11 | 6.16 | 0.57 | 0.54 | 1.06 | 0.05 | 22 | 25 |
| 3KNH_T | 99 | | 74 | 10 | 5.09 | 0.57 | -0.04 | 0.11 | 0.03 | 19 | 56 |
| 3K6Y_A | 227 | | 10 | 20 | 4.93 | 0.58 | 0.19 | 0.33 | 0.17 | 24 | 33 |
| 3G5O_B | 81 | | 63 | 10 | 0.88 | 0.58 | -0.05 | 0.56 | 0.12 | 16 | 26 |
| 1G2O_A | 262 | a/b | 29 | 19 | 5.06 | 0.59 | 0.63 | 0.51 | 0.33 | 21 | 27 |
| 2W72_A | 141 | | 74 | 27 | 3.29 | 0.62 | 0.29 | 0.33 | 0.33 | 25 | 38 |
| 2VZW_A | 146 | | 33 | 15 | 1.49 | 0.64 | 0.25 | 0.51 | 0.03 | 20 | 33 |
| 2WWO_A | 158 | | 19 | 33 | 5.56 | 0.68 | 0.29 | 0.42 | 0.22 | 32 | 45 |
| 1I9G_A | 264 | a/b | 15 | 20 | 3.18 | 0.68 | 0.76 | 0.80 | 0.30 | 16 | 23 |
| 1W5R_A | 273 | a+b | 10 | 44 | 5.38 | 0.70 | 0.39 | 0.55 | 0.28 | 30 | 34 |
| 2H7M_A | 268 | a/b | 189 | 19 | 6.82 | 0.71 | 0.61 | 0.54 | 0.05 | 15 | 16 |
| 1R88_A | 267 | a/b | 42 | 11 | 5.30 | 0.73 | 0.26 | 0.13 | 0.44 | 12 | 18 |
| 3HUG_B | 62 | | 161 | 8 | 1.33 | 0.75 | 0.10 | 0.02 | 0.19 | 19 | 14 |
| 2VLR_E | 240 | b | 23 | 33 | 10.14 | 0.76 | 0.41 | 0.32 | 0.23 | 30 | 34 |
| 2G2D_A | 160 | | 10 | 36 | 2.40 | 0.77 | 0.19 | -0.06 | 0.09 | 36 | 51 |
| 1NGK_A | 126 | a | 77 | 12 | 3.94 | 0.79 | 0.16 | 0.03 | 0.10 | 22 | 32 |
| 3R9Q_A | 233 | | 72 | 26 | 5.16 | 0.79 | 0.35 | 0.29 | 0.15 | 19 | 27 |
| 3PKA_A | 284 | | 26 | 25 | 11.45 | 0.79 | 0.43 | 0.57 | 0.34 | 25 | 32 |
| 1PM4_A | 117 | b | 88 | 7 | 11.91 | 0.81 | -0.06 | 0.34 | 0.17 | 13 | 21 |
| 2O7G_A | 88 | | 12 | 13 | 0.43 | 0.83 | 0.24 | 0.45 | 0.00 | 23 | 44 |
| 2W3G_A | 150 | | 26 | 17 | 0.50 | 0.85 | 0.45 | 0.33 | 0.06 | 27 | 36 |
| 1NFF_A | 244 | a/b | 253 | 25 | 4.88 | 0.85 | 0.37 | 0.33 | 0.34 | 24 | 34 |
| 3HST_B | 139 | | 10 | 25 | 7.38 | 0.85 | 0.28 | 1.03 | 0.29 | 24 | 33 |
| 1PZS_A | 171 | b | 19 | 24 | 8.92 | 0.87 | 0.47 | 0.52 | 0.53 | 19 | 28 |
| 1YLK_A | 163 | | 11 | 22 | 5.22 | 0.88 | 0.10 | 0.41 | 0.06 | 24 | 30 |
| 2ZFZ_A | 77 | | 36 | 13 | 1.00 | 0.89 | 0.08 | 0.21 | 0.08 | 23 | 39 |
| 3KNI_G | 181 | | 11 | 35 | 8.91 | 0.90 | 0.21 | 0.34 | 0.41 | 30 | 44 |
| 2Q7Y_B | 98 | a+b | 119 | 19 | 3.07 | 0.92 | 0.13 | 0.26 | 0.17 | 26 | 50 |
| 3G5O_A | 92 | | 12 | 9 | 1.42 | 0.92 | 0.18 | 1.09 | -0.06 | 21 | 50 |
| 3KNH_K | 119 | | 38 | 12 | 1.29 | 1.00 | -0.10 | 0.08 | -0.04 | 24 | 35 |
| 3PVV_A | 96 | | 68 | 9 | 0.36 | 1.00 | -0.20 | 0.07 | -0.07 | 20 | 32 |
| 2VLR_D | 199 | b | 19 | 56 | 8.36 | 1.04 | 0.35 | 0.26 | 0.40 | 20 | 24 |
| 2ZYQ_A | 296 | | 15 | 22 | 6.35 | 1.10 | 0.50 | 0.63 | 0.35 | 22 | 26 |
| 3QD8_A | 172 | | 27 | 19 | 5.17 | 1.11 | 0.21 | 0.25 | 0.32 | 14 | 22 |
| 1K44_A | 135 | a+b | 29 | 48 | 6.30 | 1.17 | 0.41 | 0.61 | 0.34 | 39 | 52 |
| 1LMI_A | 131 | b | 73 | 7 | 2.02 | 1.17 | 0.31 | 0.55 | 0.18 | 17 | 17 |
| 1XZ0_A | 271 | b | 17 | 30 | 17.14 | 1.19 | 0.38 | 0.68 | 0.37 | 20 | 28 |
| 3F9R_A | 246 | | 24 | 18 | 8.43 | 1.19 | 0.22 | 0.32 | 0.27 | 20 | 27 |
| 1S8N_A | 190 | a/b | 227 | 12 | 9.40 | 1.24 | 0.36 | 1.26 | 0.20 | 25 | 35 |
| 3KNH_S | 78 | | 56 | 12 | 1.97 | 1.26 | -0.14 | -0.05 | 0.16 | 26 | 32 |
| 3H87_A | 136 | | 33 | 11 | 0.60 | 1.26 | 0.60 | 1.16 | 0.07 | 25 | 47 |
| 3HNT_H | 220 | | 28 | 43 | 15.89 | 1.30 | 0.56 | 0.45 | 0.66 | 17 | 20 |
| 1F0N_A | 284 | a/b | 13 | 15 | 9.75 | 1.38 | 0.73 | 0.39 | 0.40 | 15 | 21 |
| 3KNI_7 | 48 | | 56 | 13 | 5.79 | 1.40 | -0.14 | -0.06 | 0.15 | 23 | 33 |
| 2AQ6_A | 143 | b | 16 | 15 | 5.06 | 1.40 | 0.52 | 0.98 | 0.36 | 13 | 11 |
| 3OL3_A | 98 | | 100 | 8 | 10.66 | 1.42 | 0.06 | 0.72 | -0.12 | 15 | 37 |
| 1YK3_A | 198 | a+b | 43 | 11 | 3.38 | 1.50 | 0.28 | 0.33 | 0.25 | 17 | 18 |
| 3MAY_A | 86 | | 213 | 8 | 2.48 | 1.83 | -0.12 | -0.13 | 0.09 | 20 | 31 |

| | | | | | | | | | | | |
|---|---|---|---|---|---|---|---|---|---|---|---|
| 1IDS_A | 198 | a+b | 35 | 41 | 11.17 | 1.85 | 0.39 | 0.85 | 0.54 | 25 | 32 |
| 3E3U_A | 196 | | 13 | 27 | 12.14 | 1.91 | 0.52 | 0.26 | 0.54 | 22 | 32 |
| 3HC7_A | 252 | | 56 | 9 | 17.46 | 2.03 | 0.30 | 0.42 | 0.27 | 19 | 27 |
| 2IG3_A | 127 | | 78 | 9 | 5.04 | 2.67 | 0.33 | 0.52 | 0.32 | 15 | 23 |
| 3PGX_A | 270 | | 210 | 24 | 12.42 | 2.69 | 0.73 | 1.01 | 0.41 | 25 | 36 |
| 2C92_A | 147 | | 10 | 36 | 6.59 | 2.83 | 0.40 | 0.43 | 0.51 | 22 | 29 |
| Average[f] | 133 | | 46 | 22 | 2.57 | 0.27 | 0.10 | 0.16 | 0.07 | 29 | 41 |
| Average[g] | 169 | | 51 | 23 | 3.28 | 0.29 | 0.14 | 0.18 | 0.09 | 28 | 39 |

[a]Length of protein target.
[b]SCOP type provided whenever they are available in SCOP database.
[c]The number of structural analogs used to construct the structural profile.
[d]The average sequence identity between the target sequence and the sequence of the structural analogs used for profiling.
[e]RMSD between the first I-TASSER model and the target scaffold.
[f]Average results on the cases with length <200 AA.
[g]Average results on all cases.
