# Supplementary material for: An Evolution-Based Approach to De Novo Protein Design and Case Study on Mycobacterium tuberculosis
Source: PLoS Comput Biol. 2013 Oct 24;9(10):e1003298. doi: 10.1371/journal.pcbi.1003298 (PMC3812052; doi:10.1371/journal.pcbi.1003298)
Supplement: Text S1 — Structure feature predictions and DNA/protein sequences. (PDF) [file pcbi.1003298.s011.pdf]

Text S1: Structure feature predictions and DNA/protein sequences

The sequence search simulation in the evolution-based protein design approach (EBM) uses structural profile, secondary structure, solvent accessibility, and backbone torsional angle prediction terms, in addition to the physics-based force field from FoldX [1]. A number of sophisticated methods have been developed for predicting these features which all rely on the position specific scoring matrix (PSSM) from PSI-BLAST search [2-4]. However, these methods cannot be used in our design procedure due to the high CPU cost of PSI-BLAST, as the prediction is required at each step of the Monte-Carlo simulations. In the following, we describe the method development for single-sequence based structure predictions without using PSI-BLAST.

Secondary structure prediction. The secondary structure (SS) is predicted by back propagation neural network (NN) training methods[5], which considers three states: Helix (H), Sheet (E) and Coil (C). The NN training features are based on three fingerprints of secondary structure propensity score, amino acid composition score, and BLUSOM62 substitution score[6].

Secondary structure propensity score. The SS propensity score of an amino acid type x in a particular secondary structure state h is calculated from the statistics of 45,397 non-redundant protein structures from the Protein Data Bank (PDB)[7], i.e.

$$s(x,h) = \frac{C_{x,h}/C_h}{C_x/C} \qquad \text{(S1)}$$

where C is the total number of amino acid residues in the dataset, C_x is that with amino acid type x, C_h is that in secondary structure state h, and $C_{x,h}$ is that with amino acid type x and secondary structure state h. The first three cells (Index 1-3 in Fig S2) of the fingerprints are filled with the propensity score.

Amino acid composition Score. The amino acid composition score describes the sequence environment feature of amino acids. For a given amino acid type x at position i on the training protein set, we count the frequency of occurrence of other amino acids y_i, $f(x_i,y_i)$, within a sliding window (say within ±3 residues, as shown in Figure S1). The composition score $c(x_i)$ is then calculated as the frequency multiplied by the solvent accessibility of that residue, $SA(y_i)$, in a tri-peptide (A-X-A) conformation [8], i.e.

$$c(x_i) = f(x_i, y_i)SA(y_i) \qquad \text{(S2)}$$

Thus, each position has 20 composition score corresponding to each amino acid that constitutes a fingerprint of size 20 (Index 5-24 in Fig S2).

Neural network training. The single-sequence based secondary structure prediction is trained by a one-layer neural network with fingerprint features including SS propensity score, amino acid composition score, and BLOSUM62 substitution matrix (Figure S2). The NN was trained on 5,527 non-homologous proteins, with true secondary structure assigned by DSSP [9]. Three predictors were trained using different window size of 16, 17 and 21, where in the latter two the amino acid composition score was turned off. The final SS prediction is obtained by summing up the probability score of the three predictors, where the state of the highest probability score is returned. The test on 625 proteins non-redundant to the training set shows that the overall Q3 accuracy is 69.3%.

Since the method does not run the PSI-BLAST method, it takes <<1 sec to process each sequence.

Solvent accessibility prediction. The solvent accessibility (SA) of an amino acid is categorized into three states based on the relative solvent accessibility (RSA) of the amino acid in the protein structure. An amino acid is defined as Buried (B) if RSA<0.09, or Exposed (E) if RSA>0.64, or intermediate (I) otherwise. RSA is calculated by the ratio of the actual accessibility of the amino acid in structures versus that of the amino acid in a tri-peptide conformation (A-X-A) [8].

To predict solvent accessibility, we first computed the SA propensity score of residues in the B, E and I states based on the statistics of the 45,397 non-redundant PDB structures using an equation similar to Eq. S1. The definition of amino acid composition score is same as used in SS prediction but we used 150 as normalization factor. Secondary structure information computed from our prediction method was incorporated as a binary matrix to improve the SA prediction accuracy. We assume a three state (helix, sheet and otherwise) representation of secondary structure. Therefore, the binary matrix contains three columns corresponding to each state. If an amino acid is present in a particular state the matrix value is assigned as 1, it is 0 otherwise.

The fingerprint scoring matrices in the SS training includes SA propensity (Index 1-3 of Fig S3), secondary structure prediction (Index 5-7 of Fig S3), amino acid composition score (Index 9-28 of Fig S3), and BLOSUM62 substitution matrix (Index 30-52 of Fig S3), which have been listed side-by-side and separated by noises (cells filled with black) in Figure S3.

The one-layer back propagation neural network was trained on the 5,527 non-redundant protein structures with a window size of 12. A separate test on 625 proteins shows that the average SA Q3 accuracy is 66.1%.

Backbone torsional angle prediction. The real values of backbone torsion angles (Φ and Ψ) are predicted using a similar back propagation neural-network approach as developed by Xu and Zhang [10]. The input training features include the secondary structure assignment of the target residue and the PSI-BLAST check point file, where the outputs are the real value of the torsion angles. The NN was trained on the same set of 5,527 non-homologous proteins, with true secondary structures and torsional angles were assigned by DSSP [9].

For single-sequence based torsion angle prediction, the input features of the neural network include the secondary structure as predicted by our single-sequence based NN method and the check point file that was converted from BLOSUM 62 substitute matrix. In the validation data, the method shows optimal performance with a window size of 21 for phi angle and 17 for psi angle prediction, which are selected in our predictors. A test on the same set used in the solvent accessibility benchmark of 625 non-homologous proteins, which are non-redundant to the training set, indicates that the average deviation of Φ and Ψ from the DSSP assigned experimental values are comparable to ANGLOR [4], which was trained on more computationally expensive PSI-BLAST PSSM profiles, with the deviation in Φ being only 4.3° higher than ANGLOR and Ψ only 1.5° higher.

DNA and protein sequences. The designed DNA sequences were optimized based on frequent codon usage in *E. coli* (K-12 strain). Ligation independent cloning (LIC) handles are in bold. Protein sequence lengths are given below. The N-terminal cloning residues "SNA" (lower case in sequence) remain after rTEV protease cleavage during purification, extending the length of the purified proteins by three amino acids.

DNA sequence of designed hNRNPK domain (original target scaffold, PDB ID:1ZZK):

TACTTCCAATCCAATGCACAGGGTTCTGACATCACCCTGCAGATCTCTATC
CCGACCAACATGATCGGTGCGGTTATCGGTAAAGGTGGTGAAGTTATCAAA
GAAATCCAGGAAAAAACCGGTGCGCGTATCCAGATGTCTAAACCGGAAGGT
GGTGACAAAGAAAAAATGGTTACCGTTACCGGTCCGCCGGAATCTATCGAA
AAAGCGAAAGAACTGATCATCGAAATGGTTGAAGAATCTCAGGGTCAGAAA
TTC**TAACATTGGAAGTGGATAA**

*Protein sequence of designed hnRNPK (*Length 80/83 designed/expressed)

snaQGSDITLQISIPTNMIGAVIGKGGEVIKEIQEKTGARIQMSKPEGGDKEKMVTV
TGPPESIEKAKELIIEMVEESQGQKF

DNA sequence of designed thioredoxin domain (original target scaffold, PDB ID: 1R26):

TACTTCCAATCCAATGCATCTATCGTTAAAGTTCAGTCTCCGGAAAACTTCC
AGGAAATCATCAAAGCGGGTAAACTGGTTGTTATCTACTTCTACGCGCCGTG
GTGCCCGCCGTGCCAGAAAGTTTCTCCGGAAATGGAAGCGATGGCGAAAGAA
TACGAAAACGTTATGTTCATCGCGGTTGACATCAACCACAACGAAGAACTGG
CGAAAAAATTCAACATCCAGGAACTGCCGACCATCCTGATCATCAAAGACGG
TAAAATCATGGCGTCTGTTACCGGTGCGAAACCGGAAGAAGTTTCTGAATAC
ATCTCTCAGCTGCTGCGTGAA**TAACATTGGAAGTGGATAA**

DNA sequence of designed thioredoxin (Length: 105/108 designed/expressed):

snaSIVKVQSPENFQEIIKAGKLVVIYFYAPWCPPCQKVSPEMEAMAKEYENVMF
IAVDINHNEELAKKFNIQELPTILIIKDGKIMASVTGAKPEEVSEYISQLLRE

DNA sequence of designed CISK-PO domain (original target scaffold, PDB ID: 1XTE):

TACTTCCAATCCAATGCACCGGACTCTCTGATGAAAGTTTCTATCCCGGACT
TCGAAAAAGAAGGTGAAGGTAAATCTAAACACGTTATGTACAAAATCAAAGT
TAAAACCGGTGGTGAAGAATGGGCGGTTTACCGTCGTTACTCTGACTTCTACT
GGCTGCACAAAAAACTGCAGCAGCGTTACCCGGAACTGGTTCCGGAACTGCC
GCCGAAAAAATGGATCTACTCTGCGCTGGACGAACAGATCCTGGAAAAACGT
AAACAGGGTCTGGAAAAATACATCCAGCGTATCGTTTCTCACCCGGTTCTGG
CGAACGACGAACTGGTTGTTTCTTTCCTGCAGGCGAAAGCGGAACACACCGG
T**TAACATTGGAAGTGGATAA**

Protein sequence of designed CISK-PX (Length: 116/119 designed/expressed):

snaPDSLMKVSIPDFEKEGEGKSKHVMYKIKVKTGGEEWAVYRRYSDFYWLHKK
LQQRYPELVPELPPKKWIYSALDEQILEKRKQGLEKYIQRIVSHPVLANDELVVSF
LQAKAEHTG

DNA sequence of designed Lov2 domain (original target scaffold, PDB ID:2V0U):

TACTTCCAATCCAATGCATCTTCTGGTCAGTCTCAGAACCTGGAAAACGCGG
AACAGACCTTCATCATCACCGACCCGCGTCTGCCGGACGGTCCGATCGTTTAC
GCGTCTGAAGGTTTCCTGAACCTGACCGGTTACGGTCGTGAAGAAATCCTGG
GTCGTAACTGCCGTTTCCTGCAGGGTCCGGCGACCGACCCGGCGACCGTTCA
GGAAATGCGTAACGCGCTGTCTAACGAAGAACCGTGGACCGTTGAACTGATC
AACTACAAAAAAGACGGTACCAAATTCTGGAACATCCTGACCATGGTTCCGG
TTAAAGACAACGACGGTGAAGTTATGTACTACATCGGTGTTCAGATGGACGT
TACCAAACACCGTAAAGACCGTGCGGAAGACGAAGCGATGATGTACGTTGTT
AAAACCGCGCAGGGTATCATGGAACTGATGAAAGCGATG**TAACATTGGAAG
TGGATAA**

Protein sequence of designed Luv2 (Length: 146/149 designed/expressed):

snaSSGQSQNLENAEQTFIITDPRLPDGPIVYASEGFLNLTGYGREEILGRNCRFLQ
GPATDPATVQEMRNALSNEEPWTVELINYKKDGTKFWNILTMVPVKDNDGEVM
YYIGVQMDVTKHRKDRAEDEAMMYVVKTAQGIMELMKAM

DNA sequence of designed TIF1 domain (original target scaffold, PDB ID:3IO4):

TACTTCCAATCCAATGCATTCAAAGAAATGATCGACGGTATCGTTATCCGTA
CCAACGGTAACGGTATCTTCAAAGTTGAACTGAAAAACGGTATGAAAGTTAT
GTGCCACGTTCGTGACAAAATCAAAGAAAACAAAGCGACCATCAAACCGGG
TGACTACGTTCTGGTTCGTCTGGTTCGTAAAGACCCGGTTCGTGGTACCATCA
TGGGTATCCTGGAA**TAACATTGGAAGTGGATAA**

Protein sequence of designed TIF1 (Length: 68/71 designed/expressed):

snaFKEMIDGIVIRTNGNGIFKVELKNGMKVMCHVRDKIKENKATIKPGDYVLVRLVR
KDPVRGTIMGILE

References

1. Guerois R, Nielsen JE, Serrano L (2002) Predicting changes in the stability of proteins and protein complexes: a study of more than 1000 mutations. J Mol Biol 320: 369-387.
2. Faraggi E, Zhang T, Yang Y, Kurgan L, Zhou Y (2012) SPINE X: improving protein secondary structure prediction by multistep learning coupled with prediction of solvent accessible surface area and backbone torsion angles. J Comput Chem 33: 259-267.
3. Chen H, Zhou HX (2005) Prediction of solvent accessibility and sites of deleterious mutations from protein sequence. Nucleic Acids Res 33: 3193-3199.
4. Wu S, Zhang Y (2008) ANGLOR: a composite machine-learning algorithm for protein backbone torsion angle prediction. PLoS ONE 3: e3400.
5. Rumelhart DE, Hinton GE, Williams RJ (1986) Learning representations by back-propagating errors. Nature 323: 533-536.
6. Henikoff S, Henikoff JG (1992) Amino acid substitution matrices from protein blocks. Proc Natl Acad Sci U S A 89: 10915-10919.

7. Berman HM, Westbrook J, Feng Z, Gilliland G, Bhat TN, et al. (2000) The Protein Data Bank. Nucleic Acids Res 28: 235-242.
8. Rost B, Sander C (1994) Conservation and prediction of solvent accessibility in protein families. Proteins 20: 216-226.
9. Kabsch W, Sander C (1983) Dictionary of protein secondary structure: pattern recognition of hydrogen-bonded and geometrical features. Biopolymers 22: 2577-2637.
10. Xu D, Zhang Y (2012) Ab initio protein structure assembly using continuous structure fragments and optimized knowledge-based force field. Proteins 80: 1715-1735.
